# Dissecting the Effects of Aldosterone and Hypokalemia on the Epithelial Na^+^ Channel and the NaCl Cotransporter

**DOI:** 10.3389/fphys.2022.800055

**Published:** 2022-04-26

**Authors:** Mathias Kristensen, Robert A. Fenton, Søren B. Poulsen

**Affiliations:** Department of Biomedicine, Aarhus University, Aarhus, Denmark

**Keywords:** amiloride, conns syndrome, eplerenone, mineralocorticoid receptor, thiazide-sensitive cotransporter

## Abstract

Primary hyperaldosteronism (PA) is characterized by aldosterone excess and hypertension. This may be linked to increased renal Na^+^ reabsorption *via* the epithelial Na^+^ channel (ENaC) and the NaCl cotransporter (NCC). The majority of PA patients have normal plasma K^+^ levels, but a subset of cases are associated with hypokalemia. High NCC levels observed in long-term studies with aldosterone-infused rodents have been attributed to direct effects of aldosterone. Aldosterone can also increase active phosphorylated NCC (pT58-NCC) acutely. However, direct effects of aldosterone on NCC have been contested by recent studies indicating that it is rather an indirect effect of hypokalemia. We therefore set out to determine isolated long-term aldosterone and K^+^ effects on ENaC and NCC using various *in vivo* and *ex vivo* approaches. In mice, aldosterone-induced hypokalemia was prevented by simultaneous amiloride infusion, coupled to increased cleavage of α- and γENaC but no effect on NCC. Regression analyses of *in vivo* data showed a positive correlation between aldosterone/K^+^ and αENaC but a negative correlation with NCC and pT58-NCC. *Ex vivo*, exposure of kidney tubules for 21 h to aldosterone increased cleavage of αENaC and γENaC, but no effects were observed on NCC or pT58-NCC. Exposure of tubules to low K^+^ media reduced αENaC but increased NCC and pT58-NCC. As hypokalemia can enhance cell proliferation markers in the distal convoluted tubule (DCT), we hypothesized that aldosterone infusion would increase proliferating cell nuclear antigen (PCNA) expression. Infusion of aldosterone in mice for 6 days greatly increased PCNA expression in the DCT. Collectively, *in vivo* and *ex vivo* data suggest that both aldosterone and K^+^ can increase ENaC directly. In contrast, the observed increase in abundance and phosphorylation of NCC in aldosterone-infused mice is likely an indirect effect of enhanced ENaC-mediated K^+^ secretion and subsequent hypokalemia. Thus, it is possible that NCC may only be increased in PA when the condition is associated with hypokalemia.

## Introduction

Primary hyperaldosteronism (PA), also known as Conns syndrome (Conn, 1955), is the underlying cause of high blood pressure in approximately 10% of subjects diagnosed with hypertension (Schirpenbach and Reincke, 2007; Rossi, 2010; Kayser et al., 2016; Monticone et al., 2017). It is primarily caused by bilateral idiopathic hyperplasia or aldosterone producing adenomas and is characterized by autonomous excess of circulatory aldosterone and suppression of renin (Schirpenbach and Reincke, 2007; Moraitis and Stratakis, 2011). PA may be associated with hypokalemia, and this parameter was initially used for diagnosing the disease (Kaplan, 1967; Ganguly, 1998; Buffolo et al., 2017). Later it became clear that the majority of patients with PA are normokalemic (Fagugli and Taglioni, 2011; Moraitis and Stratakis, 2011). Although the two subgroups are well-described, limited knowledge exists on how hyperaldosteronism affects renal Na^+^ reabsorption during normokalemia compared to hypokalemia.

PA is prevalently mimicked in rodents by infusing aldosterone *via* mini-pumps (Kim et al., 1998; Nielsen et al., 2007; Poulsen and Christensen, 2017; Poulsen et al., 2018). Commonly observed is that aldosterone infusion induces hypokalemia and increases kidney protein abundances of the epithelial Na^+^ channel (ENaC) and NaCl cotransporter (NCC) in the distal tubule (Kim et al., 1998; Nielsen et al., 2007; Poulsen and Christensen, 2017; Poulsen et al., 2018). ENaC and NCC are important for fine-tuning Na^+^ reabsorption, K^+^ secretion and regulating blood pressure as documented by various animal models and diseases such as Liddle’s syndrome and Pseudohypoaldosteronism Type II (increased ENaC and NCC activity may contribute to the observed hypertension) and in Pseudohypoaldosteronism Type I and Gitelman syndrome (reduced ENaC and NCC activity may cause hypotension) (Pradervand et al., 1999; Kleta and Bockenhauer, 2006; Christensen et al., 2010; Furgeson and Linas, 2010).

It was previously accepted that aldosterone stimulates ENaC and NCC *via* direct effects on the tubule segments in which they are expressed (Arroyo et al., 2011a; Arroyo et al., 2011b). However, while direct effects of aldosterone on ENaC are well-established, (Loffing et al., 2001; Gaeggeler et al., 2005; Poulsen et al., 2018), it is still debated whether long-term aldosterone excess has a direct stimulatory effect on NCC. For example, aldosterone infusion did not stimulate NCC when hypokalemia was corrected by a high K^+^ diet (Terker et al., 2016). Furthermore, NCC abundance and phosphorylation (active form) are essentially the same in mineralocorticoid receptor (MR)-negative versus MR-containing distal convoluted tubule (DCT) cells (Czogalla et al., 2016). On the other hand, acute aldosterone exposure can strongly increase cAMP independently of the MR (Haseroth et al., 1999), and because NCC is a target of cAMP, NCC may potentially be a target of aldosterone *via* an MR-independent pathway. This is supported by our recent study showing that 30 min aldosterone exposure increases cAMP in mpkDCT cells and increases NCC phosphorylation in kidney tubules *ex vivo* (Cheng et al., 2019). Similar findings are reported in studies on mDCT15 cells showing that aldosterone increases NCC phosphorylation after 12–36 h exposure (Ko et al., 2013).

Hence, the major aim of the current study was to dissect the isolated effects of aldosterone and hypokalemia on ENaC and NCC using a variety of *in vivo* and *ex vivo* approaches (see Supplementary Figure S1 for experimental design). As ENaC is critical for the kidney’s ability to reabsorb Na^+^ and secrete K^+^ (Christensen et al., 2010; Poulsen et al., 2016), we hypothesized that a combined infusion of aldosterone and the ENaC blocker amiloride in mice would prevent the aldosterone-induced drop in plasma K^+^, allowing us to investigate aldosterone effects independently of hypokalemia. Second, we studied isolated long-term effects (21 h) of aldosterone and K^+^ on ENaC and NCC *ex vivo* in kidney tubules. Third, as hypokalemia can induce cell proliferation in the DCT that may contribute to increased NCC abundance (Saritas et al., 2018), we investigated the effect of aldosterone on cell proliferation in the DCT.

## Materials and Methods

### Animal Protocols

All procedures were performed in agreement with a license issued by the Animal Experiments Inspectorate, Ministry of Food, Agriculture and Fisheries, Danish Veterinary and Food Administration.

All animal experiments were performed at Department of Biomedicine, Aarhus University, Denmark. C57BL/6JBomTac male mice (Taconic, Laven, Denmark) were housed at 27°C in metabolic cages (Tecniplast, Buguggiate, Italy) in which they had free access to water and powdered rodent chow. Three experimental protocols were applied.

*Protocol 1*. A protocol was established for infusing aldosterone. Mice were administered aldosterone [A9477; Sigma-Aldrich/Merck, St. Louis, MO, United States; 100 μg/kg per 24 h dissolved in polyethylene glycol 300 (PEG 300)] or control vehicle (PEG300) for 6 days *via* osmotic minipumps (Alzet model 1007D, Alza Corporation, Palo Alto, CA, United States). Mice were fed a powdered standard mouse chow diet [1324 (0.22% Na^+^, 0.92% K^+^), Altromin Spezialfutter GmbH & Co. KG, Large, Germany]. Aldosterone increased plasma aldosterone and reduced plasma K^+^, and increased protein abundances of αENaC and NCC (Supplementary Figure S2). The results are in line with previous studies addressing chronic effects of raised plasma aldosterone in rodents (van der Lubbe et al., 2011; Nguyen et al., 2013; Poulsen and Christensen, 2017; Poulsen et al., 2018).

Protocol 2. A protocol was established for infusing amiloride. Mice were administered amiloride (A7410; Sigma-Aldrich/Merck; 2.6 mg/kg per 24 h in PEG 300) or control vehicle (PEG 300) for 6 days *via* osmotic minipumps (Alzet model 1007D, Alza Corporation). Mice were fed a powdered standard mouse chow diet [1324 (0.22% Na^+^, 0.92% K^+^) Altromin Spezialfutter GmbH & Co. KG]. Amiloride increased plasma aldosterone, plasma angiotensin II (ANGII) and αENaC in the kidney (Supplementary Figure S3), suggesting that this dose sufficiently blocked ENaC and in response activated the renin-angiotensin-aldosterone system.

Protocol 3. Mice were administered vehicle (PEG300) on a control diet (0.22% Na^+^, 0.98% K^+^), amiloride + aldosterone (2.6 and 100 μg/kg/24 h, respectively, in PEG 300) on a control diet, or vehicle (PGE300) on a low K^+^ diet (0.22% Na^+^, 0.01% K^+^). Diets were prepared from a Na^+^, Cl^−^ and K^+^ deficient diet (C1054, Altromin Spezialfutter GmbH & Co. KG) and appropriate amounts of NaCl and K^+^ citrate were added.

### Collection and Analysis of Blood

In *protocol 1 and 3*, blood was drawn under terminal isoflurane anesthesia from the retro orbital sinus using an ammonium heparin-coated end-to-end pipette (Vitrex, Herlev, Denmark). Blood was collected in a 1.5 ml Eppendorf Tubes and immediately centrifuged at 5,000 *g* for 2 min. In *protocol 2*, blood was drawn under terminal isoflurane anesthesia *via* the portal vein using a 0.6 × 25 mm needle containing 5 µL Li^+^ heparin solution. Blood was then transferred to a 1.5 ml Eppendorf Tube and immediately centrifuged at 5,000 *g* for 2 min. Plasma aldosterone and ANGII concentrations were determined using enzyme immunoassay kits (aldosterone: EIA-5298; DRG International Inc., Springfield, NJ, United States; angiotensin II: EKE-002-12, Phoenix Pharmaceuticals, Inc., Burlingame, CA, United States). Plasma K^+^ and Na^+^ concentrations were measured using a clinical flame photometer (Model 420, Sherwood Scientific, Cambridge, United Kingdom).

### *Ex vivo* Studies on Kidney Tubule Suspensions

Male C57BL/6JBomTac mice (Taconic) were isoflurane-anesthetized and kidneys were perfused *via* the left heart ventricle with 37°C warm enzyme solution containing 1.5 mg/ml collagenase type B (Roche, Basel, Switzerland) in buffer A (125 mM NaCl, 30 mM glucose, 0.4 mM KH_2_PO_4_ 1.6 mM K_2_HPO_4_, 1 mM MgSO_4_, 10 mM Na-acetate, 1 mM α-ketoglutarate, 1.3 mM Ca-gluconate, 5 mM glycine, 48 μg/ml trypsin inhibitor, and 50 μg/ml DNase, pH 7.4). Each kidney was removed and transferred to a 2-ml Eppendorf Tube containing 37°C enzyme solution. Kidneys were cut in ∼1 mm pieces with scissors and mixed continuously at 850 rpm in a thermomixer (at 37°C, Eppendorf, Hamburg, Germany) for 5 min to facilitate detachment of tubules from one another. During the 5 min, tissue was pipetted up and down 3 × 10 times. Kidney pieces were then allowed to sediment and the supernatant containing detached tubules were transferred to a separate tube. Fresh enzyme solution was then transferred to the remaining kidney pieces and incubated at 850 rpm for 3 min and pipetted 2 × 10 times. Subsequently, detached tubules were washed 3 times in commercial cell media DMEM/F12 (L0092, biowest, Riverside, MO, United States; for aldosterone experiments) or custom-made DMEM/F12 (Terker et al., 2015) for K^+^ experiments. Finally, tubules were dissolved in 50 ml of the desired cell media and 2 ml tubules per well was transferred to 6-well plates. Tubules were then allowed to sediment whereafter media was removed and replaced by 3 ml media with the desired aldosterone and K^+^ concentrations. Tubules were then incubated at 37°C and 5% CO_2_ for 21 h. Preincubation of tubules for 21 h and subsequent exposure to 10 µM salbutamol for 2 h (Selleckchem, Houston, TX) increased pT58-NCC [Supplementary Figure S4 (Poulsen et al., 2021),], thus documenting the viability of the tubules.

### Semi-Quantitative Immunoblotting

Whole-kidney samples were prepared as described previously (Poulsen and Christensen, 2017). Kidney tubule samples were prepared by removing the cell media and adding 300 µL Laemmli sample buffer (containing 0.1 M SDS) to each well. Samples were then transferred to 1.5 ml Eppendorf Tubes, sonicated 3x5 pulses on ice, and heated to 65°C for 15 min. Semi-quantitative immunoblotting was performed as previously described (Poulsen and Christensen, 2017). In brief, membranes were incubated overnight at 4°C with the primary rabbit antibodies αENaC (Sorensen et al., 2013), γENaC [5163 (Masilamani et al., 1999; Nielsen et al., 2002)], NCC [SPC-402D, StressMarq Biosciences Inc. Victoria, BC, Canada; employed in Poulsen and Christensen (2017)] or pT58-NCC [1251 (Pedersen et al., 2010),]. Subsequently, membranes were labeled with secondary Goat Anti-Rabbit antibody (P0448, Dako, Santa Clara, CA, United States) for 1 h at room temperature. Labeling was visualized using the Enhanced Chemiluminescence system (GE Healthcare, Chicago, IL, United States) or SuperSignal West Femto Chemiluminescent Substrate (Thermo Scientific, Rockford, IL, United States). Immunoreactivity was visualized using an ImageQuant LAS 4000 imager (GE Healthcare). Densiometric analyses were performed in Image Studio Lite Ver. 5.2 (Qiagen, Hilden, Germany). Coomassie-stained gels were used to correct quantification for deviations in protein loading. The maximal deviations in total protein concentrations between samples on individual blots were ±10%. All uncropped blots and coomassies are shown in Supplementary Material.

### Immunohistochemical Labeling and Cell Counting

Paraffin-embedded kidneys from a former study were used (Poulsen and Christensen, 2017) and labeled as previously described (Poulsen and Christensen, 2017). Two-µm sections were labelled for light microscopy (NCC; SPC-402D, StressMarq Biosciences Inc.; secondary antibody: goat-anti-rabbit P0448, Dako, Glostrup, Denmark) or double-immunolabeled for confocal laser scanning microscopy (NCC; SPC-402D; StressMarq Biosciences Inc. and PCNA; P8825; Sigma-Aldrich). Imaging was performed using a Leica light microscope equipped with a digital camera (Leica, Wetzlar, Germany) and a Leica TCS SL laser scanning confocal microscope and Leica confocal software (Leica). Brightness was digitally enhanced on presented images. Percent PCNA-positive cells in DCT were assessed directly in the microscope by counting the total number of cells and PCNA-positive cells in 10 NCC-positive tubules per mouse [6 days aldosterone infusion; on average 125 nuclei per mouse (range 84–155 nuclei); tissue was obtained from Poulsen and Christensen (2017)] or 20 NCC-positive tubules per mouse [*Protocol 3*; on average 196 nuclei per mouse (range 156–251 nuclei)]. The number of NCC-positive cells per mm^2^ cortex was counted on images of a single kidney slice from each mouse. The cortical area was measured in ImageJ (Schneider et al., 2012) and was defined as the area containing glomeruli and NCC-positive tubules. On average, 485 tubules and 2,560 cells were counted per mouse.

### Statistical Analyses

Pairwise comparisons of data meeting the statistical assumptions of normality and variance homogeneity were performed using Students two-sided *t*-test, while data only meeting assumptions of normality were analyzed using Satterthwaite’s two-sided unequal variance *t*-test. If not meeting the assumptions of normality, data were ln- or square-root transformed in accordance with Sokal and Rohlf (Sokal and Rohlf, 1995) or analyzed with a Mann-Whitney *U*-test. Proportion data were logit-transformed (Sokal and Rohlf, 1995). Pairwise comparisons of more than two groups were performed using a one-way ANOVA. If not meeting the assumptions of normality and variance homogeneity, data were either ln- or square-root transformed (Sokal and Rohlf, 1995), or analyzed with a Kruskal–Wallis ANOVA on Ranks. For comparisons of more than two groups, *p*-values were adjusted for multiple comparisons using Benjamini-Yekutieli FDR correction (Narum, 2006). Line and curve fittings were performed as using linear or non-linear regression (one phase decay model). Analyses were carried out using Stata 12.0 (StataCorp, College Station, TX, United States) for Windows, SigmaPlot 12.0 (Systat Software, Inc., Chicago, Il, United States) for Windows or GraphPad Prism 7.02 (GraphPad Software, San Diego, CA, United States) for Windows. All values are presented as individual data points and mean ± SEM. All data in this paper are available on request.

## Results

### Aldosterone did Not Increase NCC When Hypokalemia was Prevented by Amiloride Infusion

**FIGURE 1:**
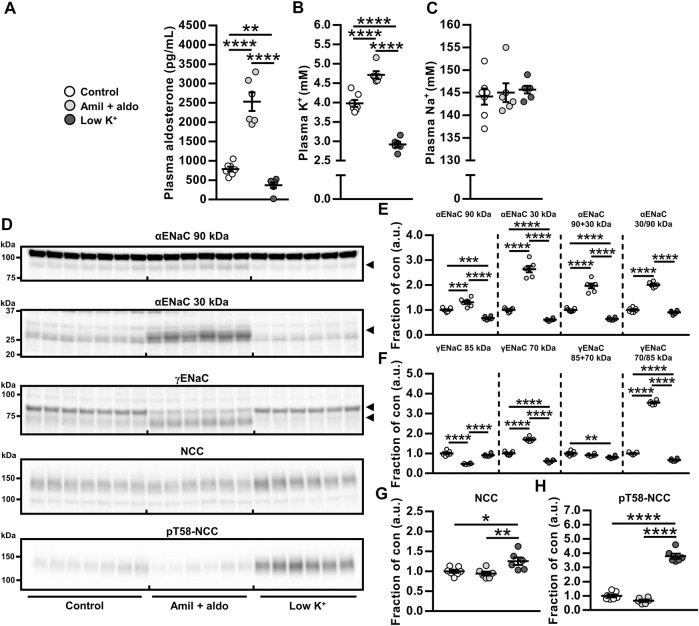
Aldosterone did not increase NCC when hypokalemia was prevented by amiloride infusion. **(A–C)** Amiloride + aldosterone (amil + aldo) infusion for 6 days increased levels of plasma aldosterone and K^+^ while a 6 days low K^+^ diet reduced plasma levels of aldosterone and K^+^. **(E,F)** Cleavages of αENaC and γENaC were increased by amiloride + aldosterone infusion but reduced by the low K^+^ diet. **(G,H)** NCC and pT58-NCC were not significantly changed in response to amiloride + aldosterone infusion but they were increased by a low K^+^ diet. Arrows in panel **(D)** indicate cleaved and non-cleaved forms of αENaC (90 and 30 kDa) and γENaC (85 and 70 kDa). Data are presented as dot plots and mean ± SEM. *n* = 7 (Control) or *n* = 6 per group (amiloride + aldosterone and low K^+^ diet). Statistical comparisons were performed using a one-way ANOVA followed by post-hoc testing with FDR-correction **(A–C** and **E–H).** *p < 0.05, **p < 0.01, ****p < 0.0001, ****p < 0.0001.

We first tested the hypothesis that the aldosterone-induced increase in NCC protein would not occur in the absence of hypokalemia. Co-administration of aldosterone and the ENaC blocker amiloride increased plasma levels of aldosterone and K^+^ whereas a low K^+^ diet reduced aldosterone and K^+^ plasma levels (protocol 3, Figures 1A,B). Thus, amiloride prevented the aldosterone-induced hypokalemia but preserved the hyperaldosteronism. Amiloride + aldosterone increased full-length αENaC (90 kDa), cleaved αENaC (30 kDa), total αENaC (90 + 30 kDa) and the ratio between cleaved and full-length αENaC (30/90 kDa) (Figures 1D,E). A low K^+^ diet in contrast reduced full-length αENaC (90 kDa), cleaved αENaC (90 kDa) and total αENaC (90 + 30 kDa) (Figures 1D,E). Amiloride + aldosterone reduced full-length γENaC (85 kDa), and increased cleaved γENaC (70 kDa) and the ratio between cleaved and full-length γENaC (70/85 kDa) (Figures 1D,F). Furthermore, the low K^+^ diet reduced cleaved γENaC (70 kDa) and the ratio between cleaved and full-length γENaC (70/85 kDa) (Figures 1D,F). NCC and phosphorylated NCC (pT58-NCC) were not significantly affected by the amiloride + aldosterone infusion when analyzing data with a one-way anova (Figures 1D,G,H). In terms of pT58-NCC, the less conservative Student’s 2-tailed *t*-test showed a significant decrease in pT58-NCC (*p* = 0.029). However, the low K^*+*^ diet increased both NCC and pT58-NCC (Figures 1D,G,H).

### Plasma Aldosterone and K^+^ Correlated Positively With Cleaved αENaC but Negatively With NCC and pT58-NCC

**FIGURE 2:**
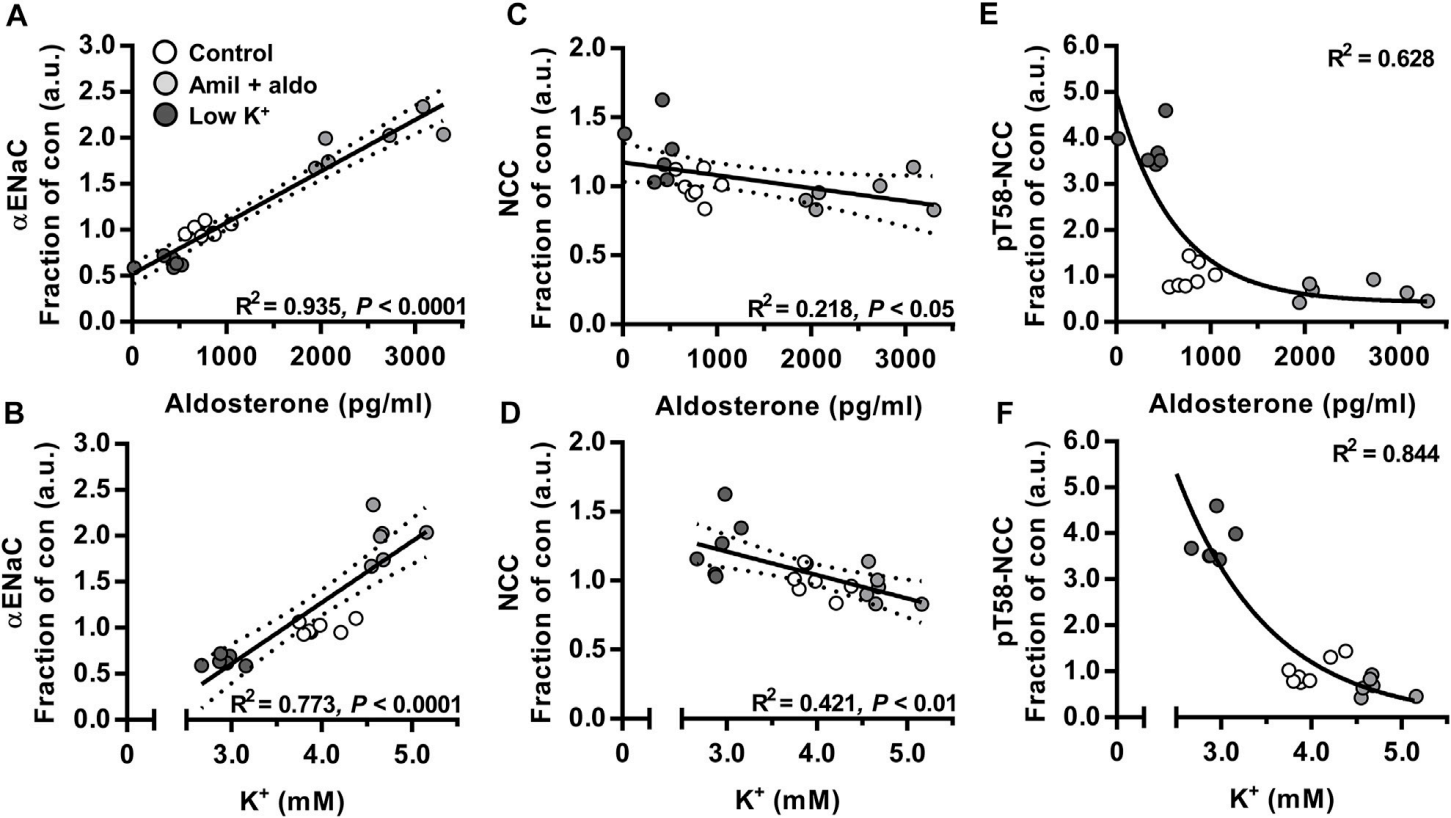
Plasma aldosterone and K^+^ correlated positively with cleaved αENaC but negatively with NCC and pT58-NCC. Data on mice that were infused with amiloride + aldosterone (amil + aldo) or fed a low K^+^ for 6 days (Figure 1) were further analyzed using regression modeling. *n* = 7 per group (Control) or *n* = 6 per group (amil + aldo and low K^+^ diet). Data were analyzed using linear **(A–D)** or non-linear regression analysis [one-phase decay model **(E,F)**].

To correlate cleaved αENaC, NCC and pT58-NCC *in vivo* over a range of aldosterone and K^+^ plasma concentrations, data presented in Figure 1 were analyzed using regression analyses. We found a positive correlation between total αENaC (30 + 90 kDa) and plasma levels of aldosterone and K^+^ (Figures 2A,B). On the contrary, for both NCC and pT58-NCC, a negative correlation was found with plasma aldosterone and K^+^ (Figures 2C–F).

### Aldosterone Increased ENaC but did Not Affect Abundance or Phosphorylation of NCC *Ex Vivo*

**FIGURE 3:**
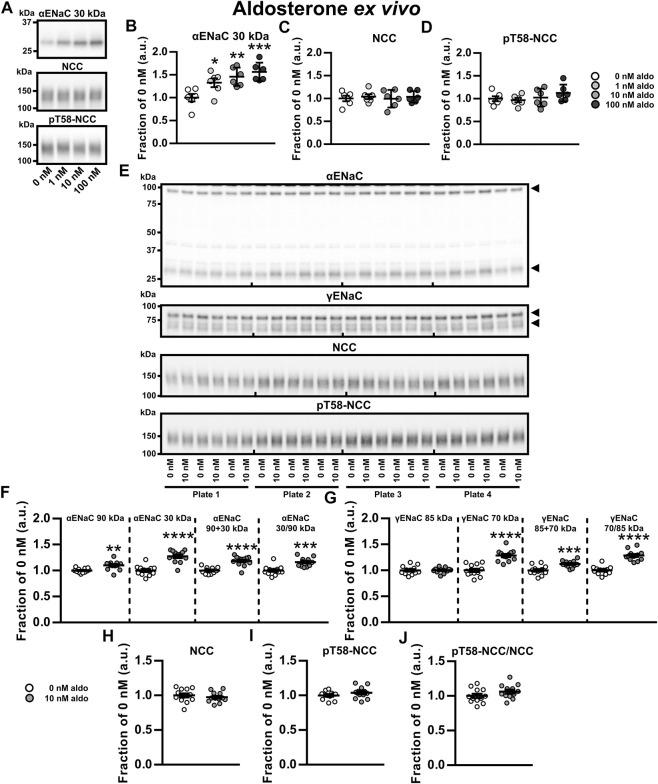
Aldosterone increased ENaC but did not affect abundance or phosphorylation of NCC *ex vivo*. **(A–D)** A dose-response *ex vivo* experiment on kidney tubule suspensions showed that exposure to physiologically relevant aldosterone (aldo) concentrations (1, 10 and 100 nM) for 21 h increased cleaved αENaC (30 kDa) whereas no significant effect was found on NCC and pT58-NCC. **(E,F)** Exposure to 10 nM aldosterone increased non-cleaved αENaC (90 kDa), cleaved αENaC (30 kDa), total αENaC (90 + 30 kDa) and the ratio between non-cleaved and cleaved αENaC (30/90 kDa). **(E,G)** Furthermore, 10 nM aldosterone increased cleaved γENaC (70 kDa), total γENaC (85 + 70 kDa) and the ratio between non-cleaved and cleaved γENaC (70/85 kDa). **(E, H–J)** No significant effect was found on NCC, pT58-NCC or the pT58-NCC/NCC ratio. Arrows in panel **E** indicate cleaved and non-cleaved forms of αENaC (90 and 30 kDa) and γENaC (85 and 70 kDa). Data are presented as dot plots and mean ± SEM. Experiments were perform in 6-well plates and quantified values were normalized to 0 nM aldosterone within individual plates. *n* = 6 per group **(A–D)** or *n* = 12 per group **(E–J).** Statistical comparisons were performed using a one-way ANOVA followed by post-hoc testing with FDR-correction **(A–D),** Satterthwaite’s 2-tailed unequal variance *t*-test [panel **(F)**, αENaC 90 kDa] or Student’s 2-tailed *t*-tests [all other comparison tests in panel **(F–J)**]. *p < 0.05, **p < 0.01, ***p < 0.001, ****p < 0.0001.

To examine direct and independent effects of aldosterone or K^+^ on ENaC, NCC and pT58-NCC, we established an *ex vivo* tubule suspension protocol allowing us to study isolated compound effects over a 21 h period. We first performed a dose-response experiment with physiologically relevant aldosterone concentrations (0, 1, 10 and 100 nM, corresponding to 360, 3,600 and 36,000 pg/ml, respectively). All tested concentrations increased cleaved αENaC (30 kDa, Figures 3A,B). However, none of the concentrations changed NCC or pT58-NCC significantly (Figures 3A,C,D).

We further assessed the effect of 10 nM aldosterone on full-length and cleaved αENaC and γENaC. Full-length αENaC (90 kDa), cleaved αENaC (30 kDa), total αENaC (90 + 30 kDa) and the ratio between cleaved and full-length αENaC were all increased by aldosterone (Figures 3E,F). No effect was observed on full-length γENaC (85 kDa) (Figures 3E,G), but cleaved γENaC (70 kDa), total γENaC (85 + 70 kDa) and the ratio between cleaved and full-length γENaC (70/85 kDa) were increased by aldosterone (Figures 3E,G). Once again, 10 nM aldosterone did not significantly affect abundances of NCC and pT58-NCC (Figures 3E,H–J). Similar findings on αENaC and NCC were observed in an additional experiment with a higher sample size (*N* = 30 per group) (Supplementary Figure S5). Together, this data indicate that *ex vivo* exposure of kidney tubules to aldosterone increases the abundance and the cleavage of α- and γENaC but has no effect on NCC.

### Low K^+^ Reduced αENaC but Increased NCC and pT58-NCC *Ex Vivo*

**FIGURE 4:**
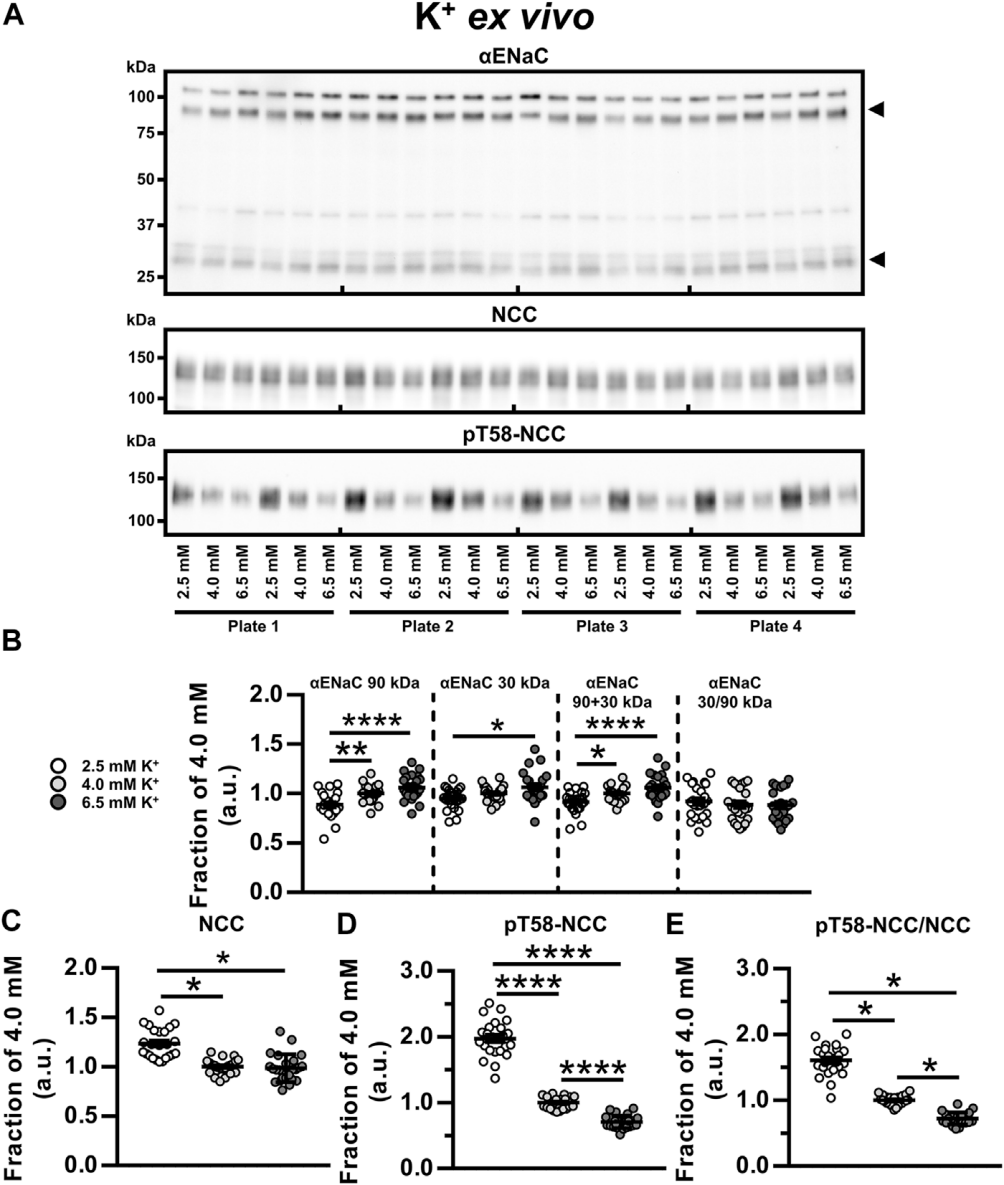
Low K^+^ reduced αENaC but increased NCC and pT58-NCC *ex vivo*. **(A, B)** Semiquantitative immunoblotting showed that *ex vivo* exposure of kidney tubule suspensions for 21 h to 2.5 mM K^+^, as compared to 6.5 mM K^+^, significantly reduced non-cleaved αENaC (90 kDa), cleaved αENaC (30 kDa) and total αENaC (90 + 30 kDa). **(A,C)** Exposure to 2.5 mM K^+^ increased NCC compared to 4.0 and 6.5 mM K^+^. **(A,D,E)** Exposure to 2.5 mM K^+^ increased pT58-NCC and the pT58-NCC/NCC ratio compared to 4.0 and 6.5 mM K^+^. Moreover, 6.5 mM K^+^ reduced pT58-NCC and the pT58-NCC/NCC ratio compared to 4.0 mM K^+^. Experiments were perform in 6-well plates and quantified values were normalized to 4.0 mM K^+^ within individual plates. Data from three individual experiments were pooled. Data are presented as dot plots and mean ± SEM. *n* = 24 per group. Statistical comparisons were performed using a one-way ANOVA followed by post-hoc testing with FDR-correction (αENaC 90 kDa, αENaC 90 + 30 kDa, αENaC 30/90 kDa and pT58-NCC) or Kruskal–Wallis ANOVA on Ranks (αENaC 30 kDa, NCC and pT58-NCC/NCC). *p < 0.05, **p < 0.01**, ****p < 0.0001.

To examine the isolated effects of K^+^, kidney tubules were exposed to 2.5, 4.0 and 6.5 mM K^+^ *ex vivo* for 21 h. Compared to 4.0 and 6.5 mM K^+^, exposure to 2.5 mM K^+^ reduced full-length αENaC (90 kDa) and total αENaC (90 + 30 kDa) (Figures 4A,B). Furthermore, cleaved αENaC (30 kDa) was reduced by 2.5 mM compared to 6.5 mM K^+^ (Figures 4A,B). NCC and pT58-NCC were significantly increased by 2.5 mM K^+^ compared to 4.0 mM K^+^ (Figures 4A,C–E). In the 21 h timeframe, 6.5 mM K^+^ did not significantly alter NCC compared to 4.0 mM K^+^, whereas it significantly reduced pT58-NCC (Figures 4A,C–E).

### Aldosterone Infusion for 6 days Increased the Proliferation Marker PCNA in Renal DCT

**FIGURE 5:**
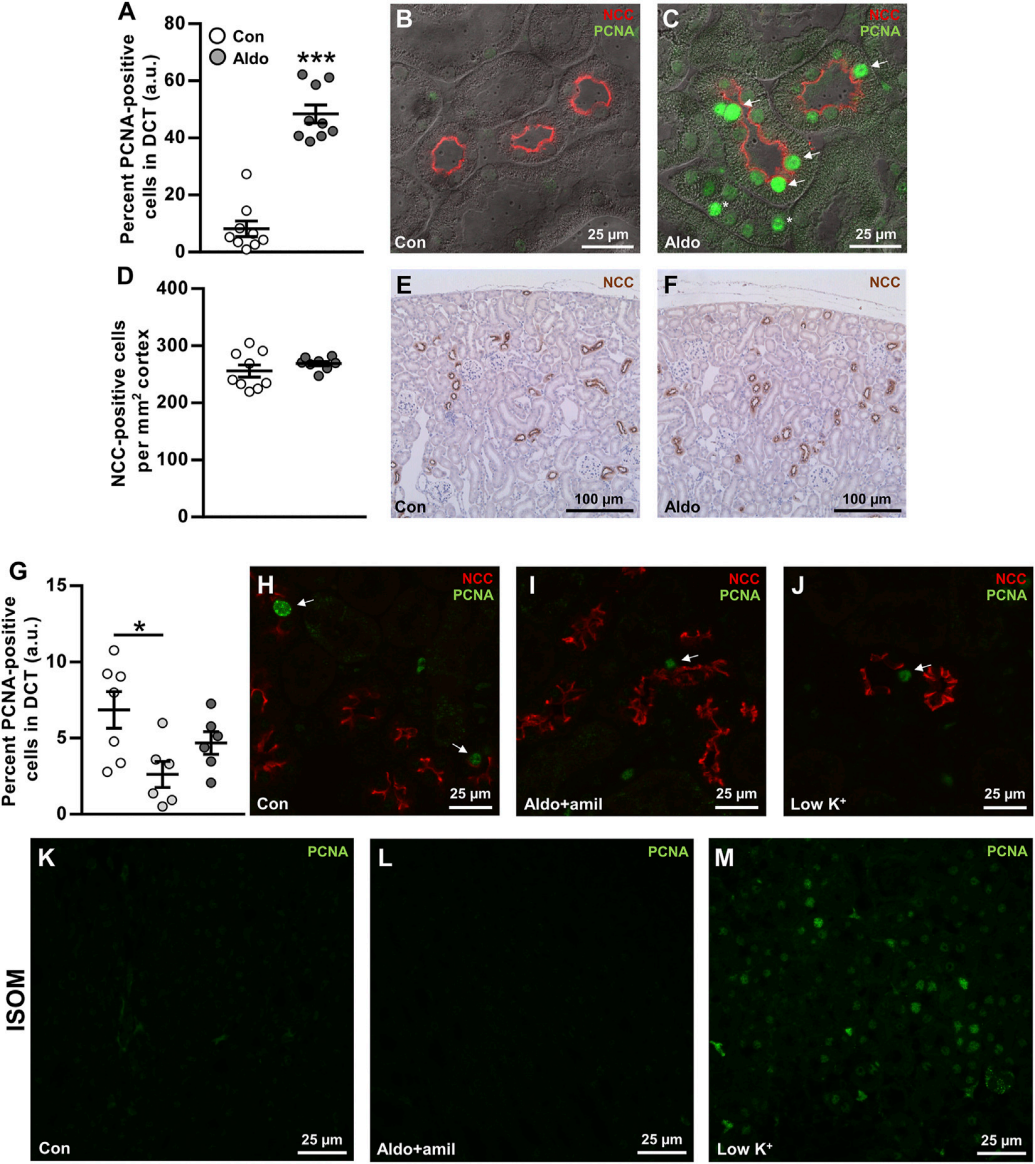
Aldosterone infusion for 6 days increased the proliferation marker PCNA in renal DCT. **(A–C)** The percent PCNA-positive cells in the DCT was markedly increased by 6 days aldosterone infusion. Arrows indicate PCNA in DCT cells. Asterisks indicate PCNA in non-DCT cells. **(D–F)** The numbers of DCT cells/mm^2^ cortex were not changed significantly by 6 days aldosterone infusion. **(G–J)** Amiloride + aldosterone infusion for 6 days reduced the number of PCNA-positive DCT cells, whereas no significant effect was found in mice fed a low K^+^ diet for 6 days. Arrows indicate PCNA in DCT cells. **(K–M)** In the inner stripe of the outer medulla (ISOM), qualitative assessment showed greater PCNA labeling in mice fed a low K^+^ diet whereas no clear effect was observed in the mice infused with amiloride + aldosterone. Kidney tissue in panel **(A–F)** originated from a previously published study (Poulsen and Christensen, 2017). Data are presented as dot plots and mean ± SEM. *n* = 9 [control and aldosterone **(A–F)**], *n =* 7 [control **(G–M)**] or *n =* 6 [amiloride + aldosterone and low K^+^ **(G–M)**]. Statistical comparisons were performed using Satterthwaite’s 2-tailed unequal variance *t*-test **(A,D)** or a one-way ANOVA followed by post-hoc testing with FDR-correction **(G)**. *p < 0.05, ***p < 0.001.

Hypokalemia has previously been associated with increased cell proliferation in the DCT after 3 days (Saritas et al., 2018) and increased DCT length after 4 weeks (Su et al., 2020). We hypothesized that similar effects would occur after infusion with aldosterone. Sections from a previous study (Poulsen and Christensen, 2017), in which mice had developed frank hypokalemia in response to 6 days aldosterone infusion, were fluorescence-labeled with NCC (DCT cell marker) and the proliferation marker, PCNA. DCT cells positive for PCNA were markedly increased in aldosterone-infused mice (Figures 5A–C), but there was no significant effect on the number of DCT cells per mm^2^ cortex (Figures 5D–F).

To study the effects of aldosterone on PCNA expression in the DCT in a condition without hypokalemia, we labeled sections from mice that had been infused with amiloride + aldosterone or fed a low K^+^ diet for 6 days (*Protocol 3*). The amiloride + aldosterone infusion reduced the number of PCNA-labeled DCT cells, but no significant effect was observed in mice fed the low K^+^ diet (Figures 5G–J). However, when assessing the inner stripe of the outer medulla (ISOM), PCNA expression was clearly increased by the low K^+^ diet.

## Discussion

**FIGURE 6:**
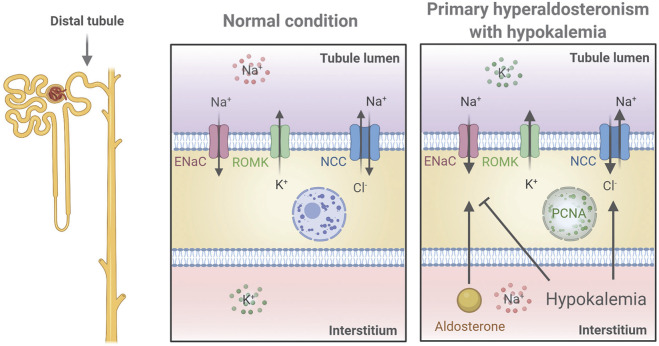
Proposed mechanistic regulation of NCC and ENaC in the renal distal tubule in a mouse model of primary hyperaldosteronism associated with hypokalemia. Aldosterone stimulates ENaC to increase Na^+^ reabsorption thus promoting K^+^ secretion *via* ROMK leading to hypokalemia. Hypokalemia stimulates NCC and furthermore works as negative feedback to aldosterone by inhibiting ENaC. Finally, increased plasma aldosterone increases PCNA in the nucleus indicating cell proliferation, but whether this occurs directly or indirectly *via* stimulation of DCT cells was not determined. The figure was created with BioRender.com.

Various studies have reported contrasting data on whether aldosterone has a direct effect on the DCT to increase NCC (Ko et al., 2013; Czogalla et al., 2016; Terker et al., 2016; Cheng et al., 2019). To investigate this further, we used *in vivo* and *ex vivo* approaches to study K^+^-independent/isolated long-term effects of aldosterone on NCC. Our data showed that blocking ENaC during aldosterone excess prevented the development of hypokalemia and the observed increase in NCC when aldosterone was administered alone. Testing the isolated effect of aldosterone *ex vivo* supported that aldosterone had no long-term effect on NCC and that low K^+^, independently of aldosterone, could increase the abundance and phosphorylation of NCC. This effect may be mediated *via* basolateral K^+^ channels because the stimulatory effect of low K^+^ on NCC is absent in Kir4.1 and Kir5.1 KO mice (Malik et al., 2018; Wang et al., 2018). Normally, high levels of glucocorticoids are thought to occupy the MR and prevent aldosterone from exerting a regulatory effect in the DCT1, which lacks the glucocorticoid inactivating enzyme, 11β-hydroxysteroid dehydrogenase 2. However, even in our *ex vivo* setting, without potential occupancy of the MR by glucocorticoids, no effects of aldosterone on NCC were observed within a 21 h period. In contrast, both α- and γENaC were clearly regulated by aldosterone *ex vivo*. Collectively, our data support that the stimulatory effect of long-term aldosterone excess on NCC is due to secondary effects of reduced plasma K^+^ (Figure 6).

A question is why *ex vivo* aldosterone exposure of kidney tubules increases NCC phosphorylation acutely (Cheng et al., 2019) but not long-term. Similarly to aldosterone, we have recently shown in kidney tubules that the β2-adrenergic receptor agonist salbutamol increases NCC phosphorylation acutely (Poulsen et al., 2021). However, exposing tubules to salbutamol for 24 h did not affect NCC abundance or phosphorylation (Supplementary Figure S6), but yet, after keeping tubules in control media for 21 h they still responded to salbutamol by increasing NCC phosphorylation (Supplementary Figure S4). Why is there this deviation between acute and chronic stimulation of receptors mediating NCC phosphorylation? One possible explanation is that the effects of hormones may wane rapidly despite continues exposure. For example, the β2-adrenergic receptor can be desensitized due to uncoupling of the receptor from the G protein resulting in reduced stimulation of cAMP production (Hausdorff et al., 1990), potentially explaining why the salbutamol effect on NCC phosphorylation was transient. Since aldosterone can increase cAMP independently of the MR (Haseroth et al., 1999), a similar mechanism may exist for aldosterone, which could explain the diverging effect of acute and long-term exposure on NCC phosphorylation. Thus, it is possible that the role of aldosterone in the DCT mainly is to adjust NaCl reabsorption acutely. It should be highlighted that *in vitro* exposure of mDCT15 cells to aldosterone for 12–36 h increased NCC phosphorylation (Ko et al., 2013), but why there are discrepancies between aldosterone effects *ex vivo* and *in vitro* is unclear.

Our *ex vivo* tubule studies also extend the current knowledge on how ENaC is regulated. Previous studies addressing how aldosterone regulates abundance and proteolytic activation/cleavage of ENaC are limited to *in vivo* and cell system studies (Rossier and Stutts, 2009). As mentioned, *in vivo* infusion of aldosterone in mice changes a number of physiological parameters including plasma K^+^ and Na^+^ levels (Poulsen and Christensen, 2017). Here, we show that both the abundances and cleavage of α- and γENaC are increased directly by aldosterone in native kidney tubules *ex vivo*. Furthermore, we show that K^+^ in itself can increase the abundance of αENaC, building on the recent observations that ENaC is directly regulated by K^+^ independently of aldosterone. This includes studies on Kir4.1 KO mice, which have increased expression of ENaC subunits (Su et al., 2016), and on cell studies showing that the stimulatory effect of K^+^ on ENaC currents may depend on Kir4.1/5.1 (Sorensen et al., 2019). The K^+^-induced increase in αENaC found in the present study may occur *via* a similar mechanism.

Six days aldosterone infusion markedly increased the expression of the cell proliferation marker, PCNA, in the DCT. Similarly, Saritas et al. (2018) showed an increase in the proliferation marker Ki-67, in the DCT of mice fed a low K^+^ diet for 3 days. To test if the increase in PCNA expression by aldosterone could be secondary to hypokalemia, we further assessed kidneys from mice that were infused with amiloride + aldosterone (had mild hyperkalemia) and mice that were fed a low K^+^ diet for 6 days (were hypokalemic). PCNA expression in the DCT was significantly reduced by amiloride + aldosterone infusion. A possible explanation for this could be that K^+^ channels are involved in controlling cell-cycle progression and that this was influenced by the high plasma K^+^ levels (Urrego et al., 2014). In the low K^+^ group, PCNA expression was clearly increased in the ISOM, but in contrast to Saritas et al. (2018), we did not observe any significant effect on cell proliferation in the DCT. While this finding was unexpected, it is possible that the effects of a low K^+^ diet on cell proliferation are different at 3 days relative to 6 days. This is supported by that PCNA expression is markedly greater in mice fed a high K^+^ diet for 3 days compared to 14 days (Cheval et al., 2004). Thus, cell proliferation may be most pronounced during the initial face after shifting animals to a low K^+^ diet. How does that correlate with the greater PCNA expression observed after 6 days of aldosterone infusion in our study? Perhaps aldosterone infusion, in contrast to a low K^+^ diet, does not affect plasma K^+^ levels for the first couple of days. Thus, the 6 days aldosterone infusion might be more comparable to a 3-days low K^+^ diet. A final possibility is that, by using the proliferation marker PCNA that is maximal expressed during the S phase (Nemoto et al., 1993), we do not detect cells in the G1, G2, or M-phase that would be detected using Ki-67 (Saritas et al., 2018).

Saritas et al. (2018) did not quantify the length of the DCT in response to a low K^+^ diet. In a more recent study it was however reported that a low K^+^ diet increased the DCT length by 13% after 4 weeks of low K^+^ diet (Su et al., 2020). We did not observe a significant increase in the number of DCT cells per mm^2^ cortex after 6 days aldosterone infusion, but it is possible that a longer infusion time was required in order to have an effect. However, increased PCNA labeling was not limited to the DCT but was also observed in other cortical tubules. Thus, it cannot be excluded that the whole cortical region proliferated and therefore masked a potential increase in the DCT length. In a clinical context, it has not been investigated if renal cell proliferation is changed in patients with PA and whether this could contribute to the etiology of the disease.

Our study indicates that high NCC levels in rodent models with high aldosterone are most likely a response to hypokalemia occurring as a result of aldosterone enhanced ENaC activity. This is in line with what is reported in other studies (Terker et al., 2016). How can this knowledge contribute clinically? We speculate that renal NCC protein abundance is only increased by aldosterone excess if it is associated with hypokalemia. An obvious strategy to lower BP during aldosterone excess could be to reduce Na^+^ reabsorption using thiazide treatment. However, reducing NCC activity with thiazide may increase Na^+^ delivery to ENaC-expressing segments and enhance K^+^ secretion (McDonough and Youn, 2017), lowering plasma K^+^ even more. Therefore, ENaC inhibitors, such as benzamil and amiloride, could be better options for lowering BP under this condition as they could reduce Na^+^ reabsorption *via* ENaC, normalize plasma K^+^ and ultimately normalize NCC. This basic experimental proposal needs to be tested further. However, it is in line with current clinical therapy strategies using ENaC inhibitors for reducing renal Na^+^ retention and treating hypertension in PA (Cobb and Aeddula, 2021).

## Data Availability

The original contributions presented in the study are included in the article/Supplementary Material, further inquiries can be directed to the corresponding author.
