# Supplementary material for: Dissecting the Effects of Aldosterone and Hypokalemia on the Epithelial Na+ Channel and the NaCl Cotransporter
Source: Front Physiol. 2022 Apr 26;13:800055. doi: 10.3389/fphys.2022.800055 (PMC9086401; doi:10.3389/fphys.2022.800055)

Supplemental Figures

Dissecting the effects of aldosterone and hypokalemia on the Epithelial Na^+ channel (ENaC) and the NaCl cotransporter (NCC)

SUPPLEMENTAL FIGURE S1 | Schematic outline of *in vivo* and *ex vivo* experimental protocols............ 2

SUPPLEMENTAL FIGURE S2 | Effects of 6 days aldosterone infusion on αENaC, γENaC and NCC. 3

SUPPLEMENTAL FIGURE S3 | Effects of 6 days amiloride infusion αENaC, γENaC and NCC............... 4

SUPPLEMENTAL FIGURE S4 | Kidney tubules were viable ex vivo after 21 hrs. 5

SUPPLEMENTAL FIGURE S5 | *Ex vivo* aldosterone exposure increased cleaved αENaC but did not regulate NCC and pT58-NCC. 6

SUPPLEMENTAL FIGURE S6 | Short and longterm exposure to the β2-adrenergic receptor agonist salbutamol. 7

UNCROPPED WESTERN BLOT IMAGES AND COOMASSIE GELS... 8

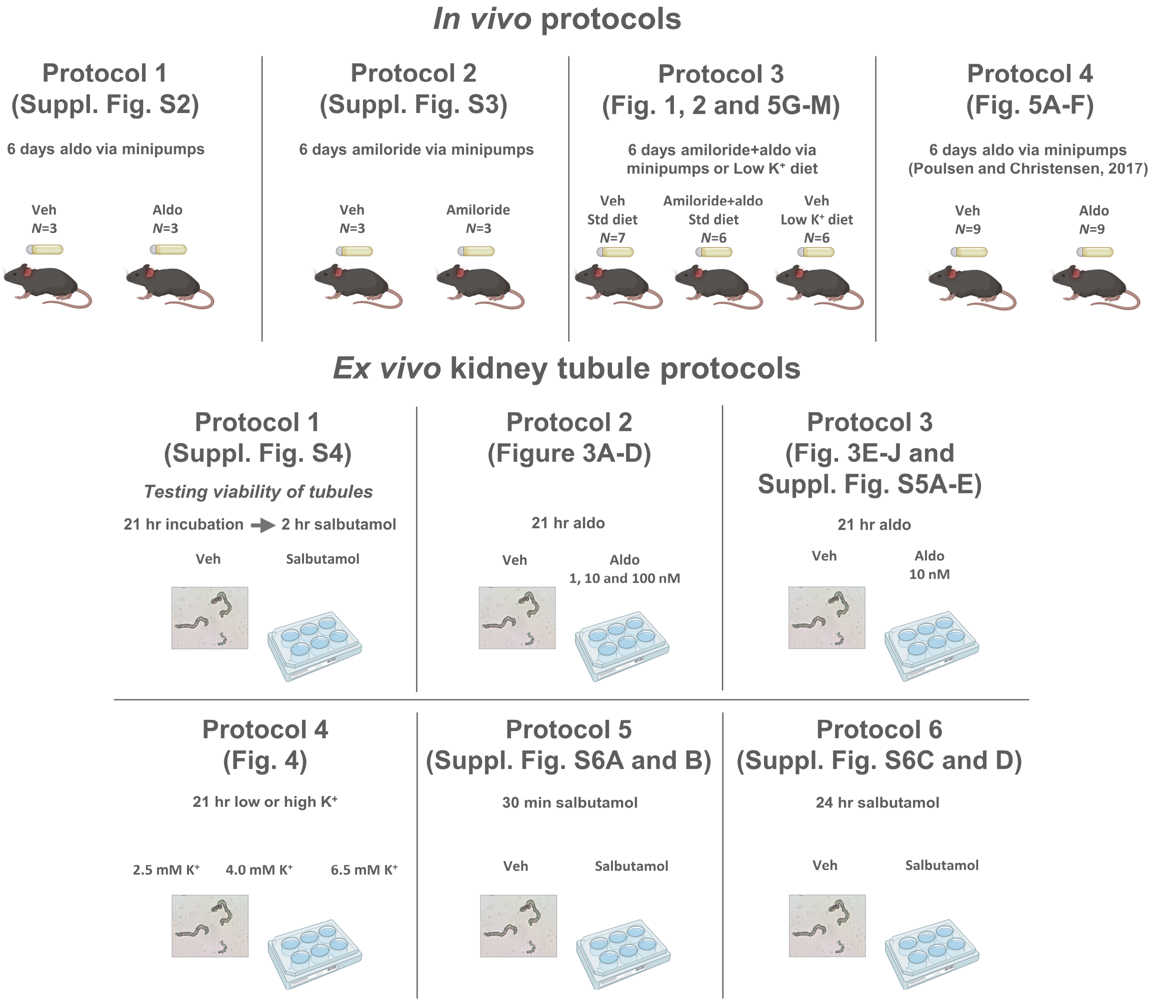

SUPPLEMENTAL FIGURE S1 | Schematic outline of *in vivo* and *ex vivo* experimental protocols. The figure was created with BioRender.com

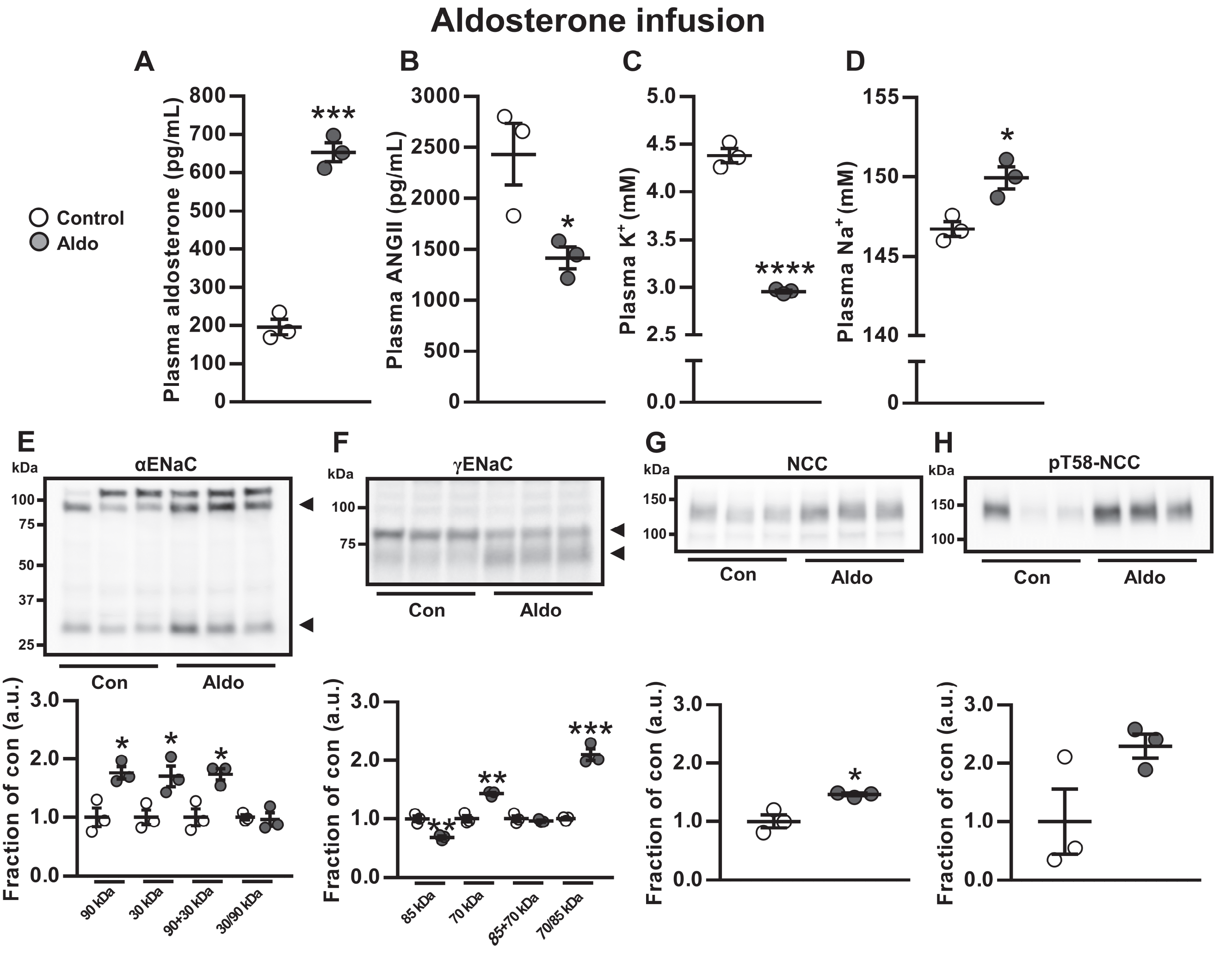

SUPPLEMENTAL FIGURE S2 | Effects of 6 days aldosterone infusion on αENaC, γENaC and NCC. **(A-D)** Aldosterone infusion increased plasma aldosterone, reduced plasma ANGII, reduced plasma K^+ and increased plasma Na^+. **(E-G)** Semiquantitative immunoblotting showed that aldosterone infusion increased abundance and cleavage of αENaC, cleavage of γENaC and abundance of NCC, while it tended to increase pT58-NCC (*P* = 0.094). Data are presented as dot plots and mean ± SEM. *N* = 3 per group. Statistical comparisons were performed using Student's 2-tailed *t*-tests or a Mann-Whitney *U*-test (αENaC 90+30 kDa). **P* < 0.05, ***P* < 0. 01, ****P* < 0.001, *****P* < 0.0001.

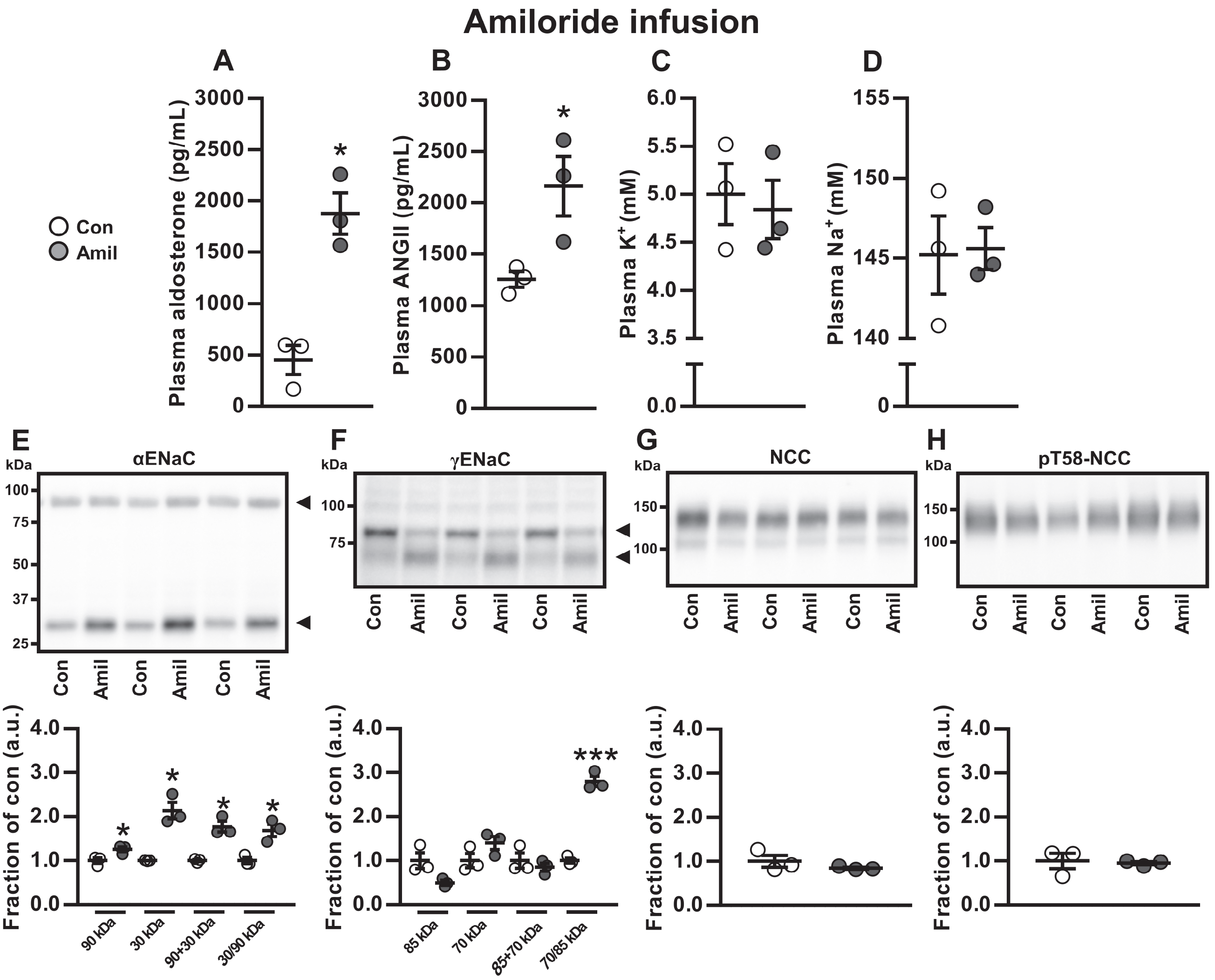

SUPPLEMENTAL FIGURE S3 | Effects of 6 days amiloride infusion on αENaC, γENaC and NCC. **(A-D)** Amiloride infusion increased plasma aldosterone and plasma ANGII. No significant effect of amiloride was found on plasma K$^+$ and plasma Na$^+$. **(E-G)** Semiquantitative immunoblotting showed that amiloride infusion significantly increased cleavage of α- and γENaC but no significant effect was found NCC (P = 0.324) or pT58-NCC (P = 0.800). Data are presented as dot plots and mean ± SEM. N = 3 per group. Statistical comparisons were performed using Student's 2-tailed t-tests (ANGII, K$^+$, Na$^+$, αENaC 90, γENaC 85 kDa, γENaC 70 kDa, γENaC 70/85 kDa, NCC and pT58-NCC), Satterthwaite's two-sided unequal variance t-test (αENaC 30 kDa) or Mann-Whitney U-test (aldosterone, αENaC 90 + 30 kDa, αENaC 30/90 kDa and γENaC 85 + 70). *$P < 0.05$, ***$P < 0.001$.

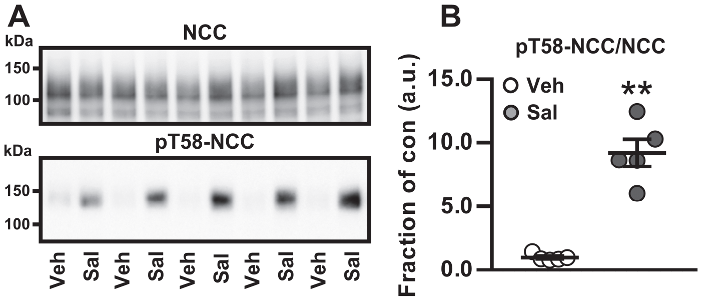

SUPPLEMENTAL FIGURE S4 | Kidney tubules were viable *ex vivo* after 21 h. To test the viability of DCT cells in tubule suspensions that had been pre-incubated for 21 h in cell media, tubules were stimulated with the β2-adrenergic receptor agonist salbutamol (Sal) for 2 h. Salbutamol clearly increased NCC phosphorylation on T58 compared to vehicle (Veh) indicating that the tubules were viable. Data are presented as dot plots and mean ± SEM. $N = 5$ per group. Statistical comparisons were performed using Satterthwaite's two-sided unequal variance *t*-test. $^{**}P < 0.01$.

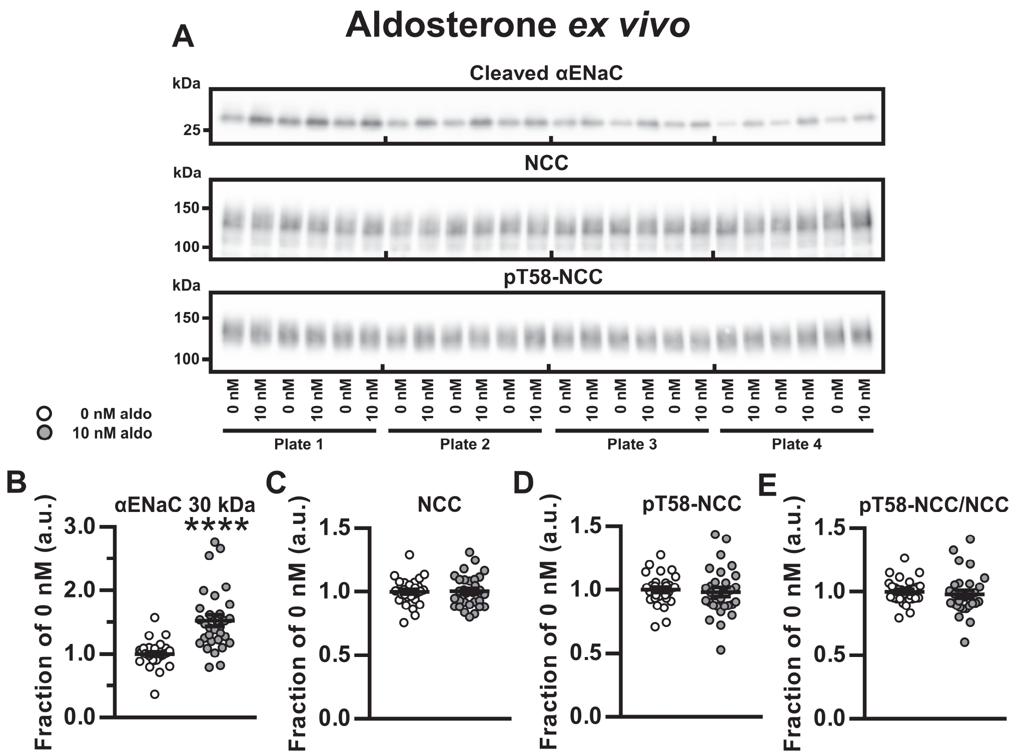

SUPPLEMENTAL FIGURE S5 | *Ex vivo* aldosterone exposure increased cleaved αENaC but did not regulate NCC and pT58-NCC. **(A)** Exposure to 10 nM aldosterone increased cleaved αENaC but had no significant effect on NCC, pT58-NCC or the pT58-NCC/NCC ratio. Data are presented as dot plots and mean ± SEM. Experiments were perform in 6-well plates and quantified values were normalized to 0 nM aldosterone within individual plates. N = 30 per group (data from three individual experiments were pooled). Statistical comparisons were performed using Mann-Whitney U-test (panel **A and B**, αENaC 30 kDa), Student's 2-tailed t-tests (panel **A and C**, NCC) or Satterthwaite's 2-tailed unequal variance t-test (panel **A, D and E**; pT58-NCC and pT58-NCC/NCC). ****$P < 0.0001$**.**

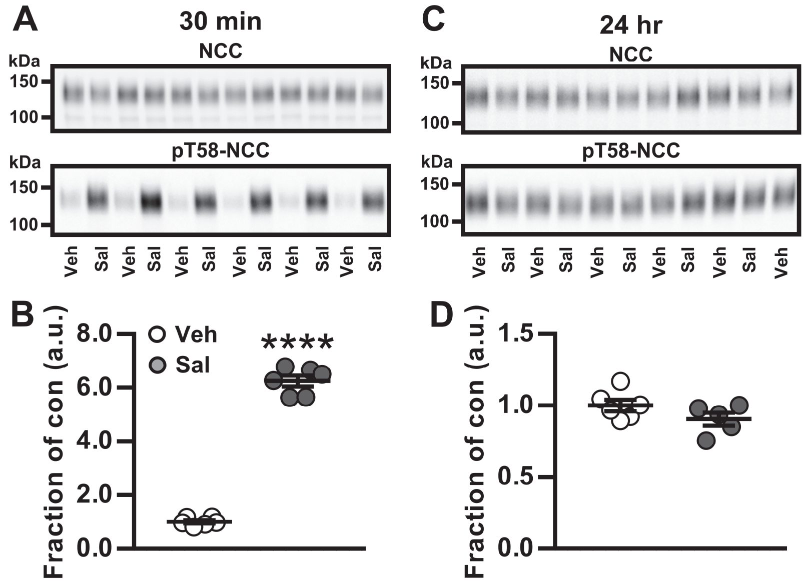

SUPPLEMENTAL FIGURE S6 | Short and long-term exposure to the β2-adrenergic receptor agonist salbutamol. **(A)** *Ex vivo* exposure of kidney tubules to the β2-adrenergic receptor agonist salbutamol (Sal) for 30 min increased NCC phosphorylation compared to vehicle (Veh). **(B)** In contrast, 24 h exposure had no effect. Data are presented as dot plots and mean ± SEM. N = 5-6 per group. Statistical comparisons were performed using Satterthwaite's two-sided unequal variance t-test (panel **A and B**) or Student's 2-tailed t-test (**panel C and D**). ****$P < 0.0001$.

UNCROPPED WESTERN BLOT IMAGES AND COOMASSIE GELS

Supplemental Figure S2

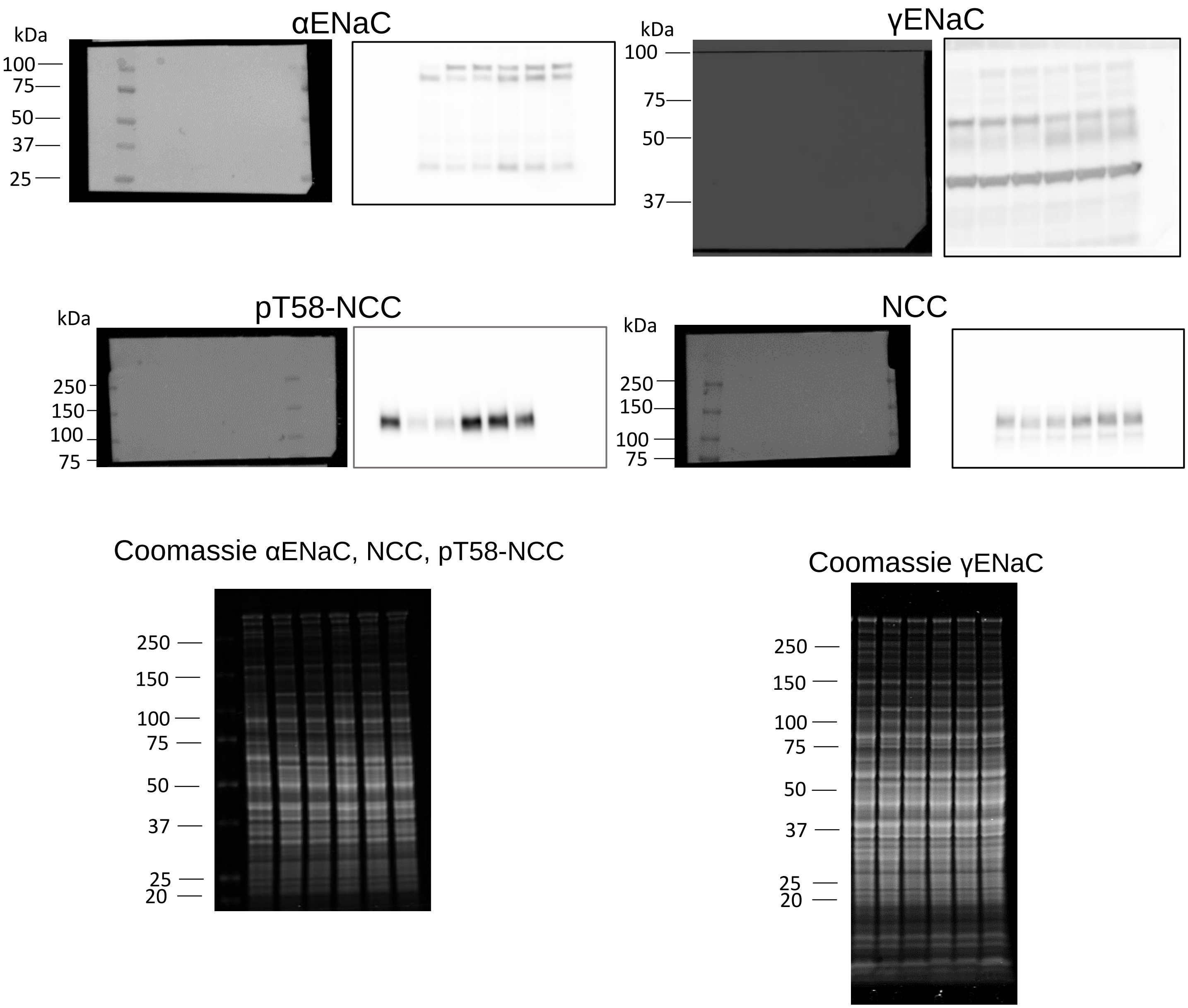

Supplemental Figure S3

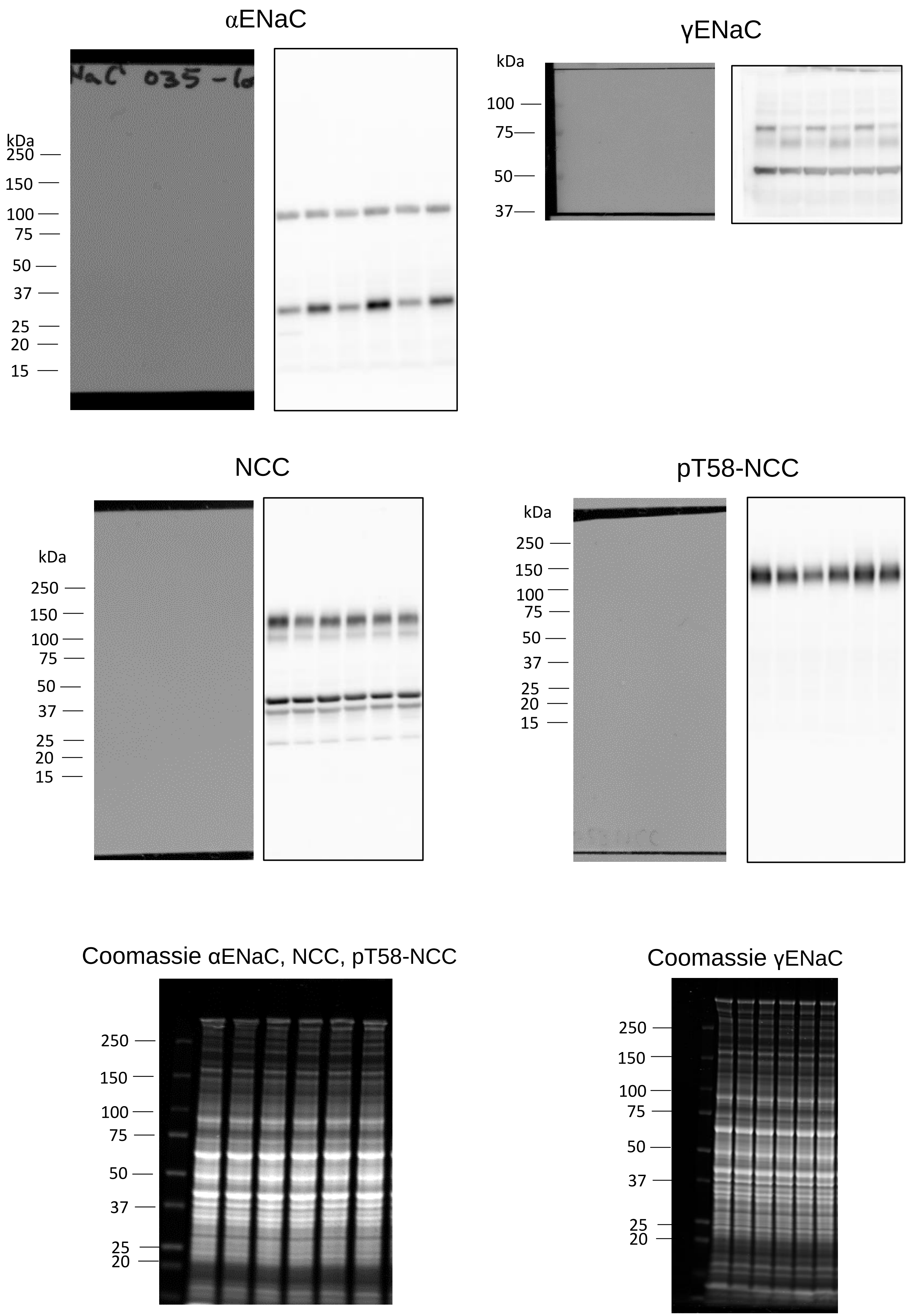

Supplemental Figure S4

NCC

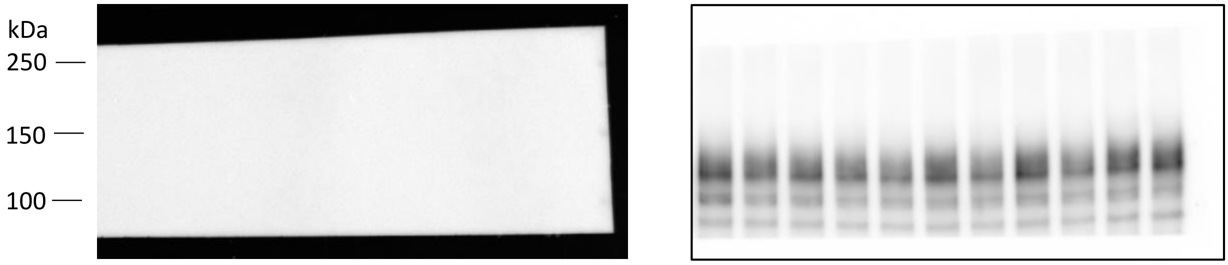

pT58-NCC

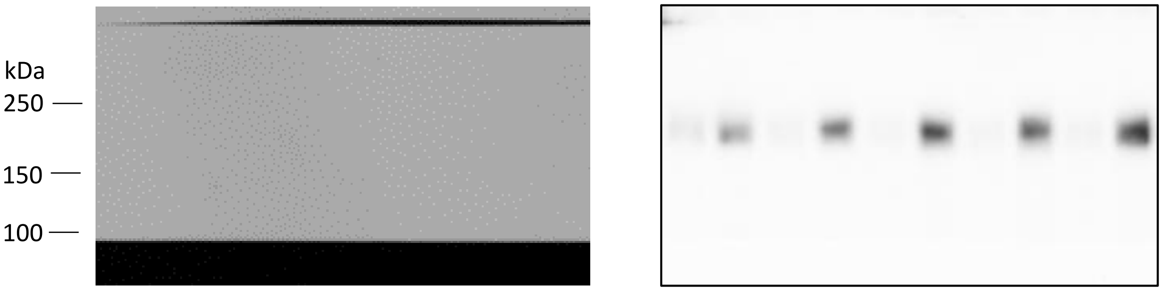

Coomassie for all blots

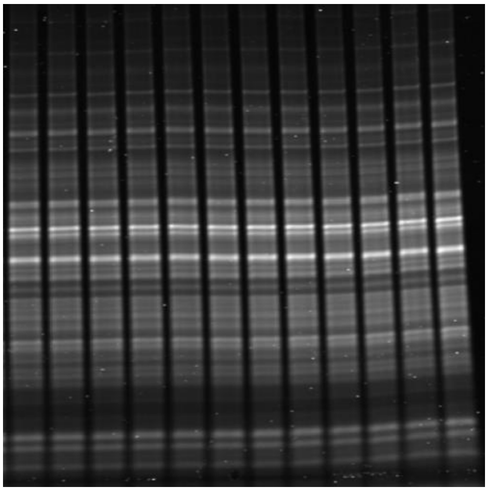

Figure 1

αENaC 90 kDa

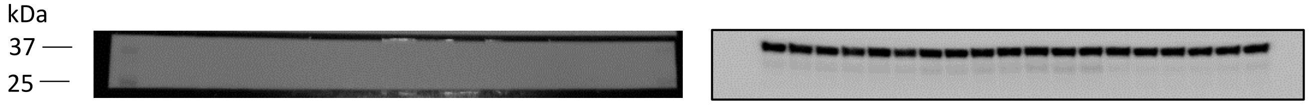

αENaC 30 kDa

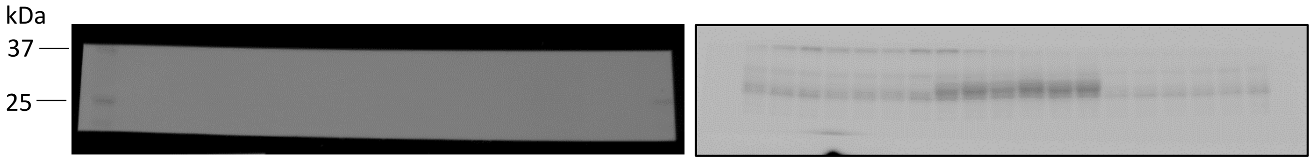

γENaC

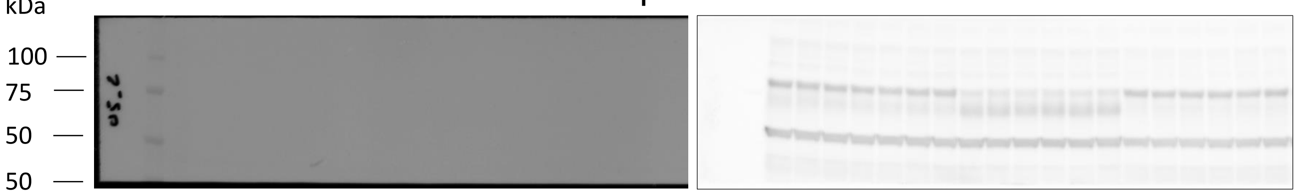

NCC

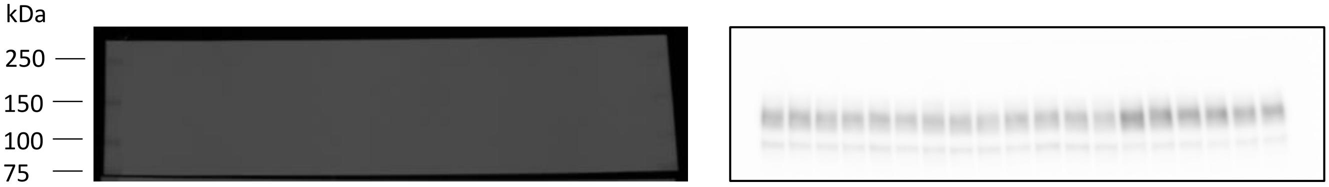

pT58-NCC

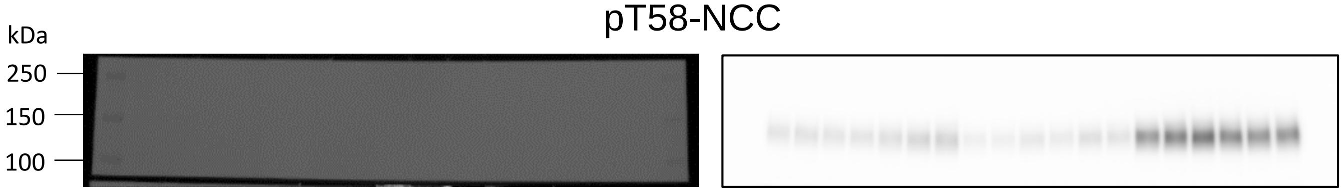

Coomassie αENaC 90, αENaC 30 kDa, NCC and pT58-NCC

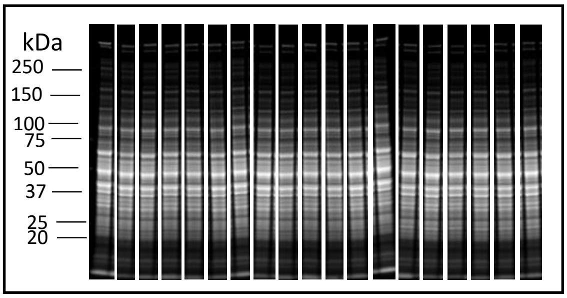

Samples were loaded in a different order on the coomassie gel compared to the western blots. Image of the coomassie gel has been edited to present the loading order used for western blotting. The presented samples originate from the same image.

Coomassie γENaC

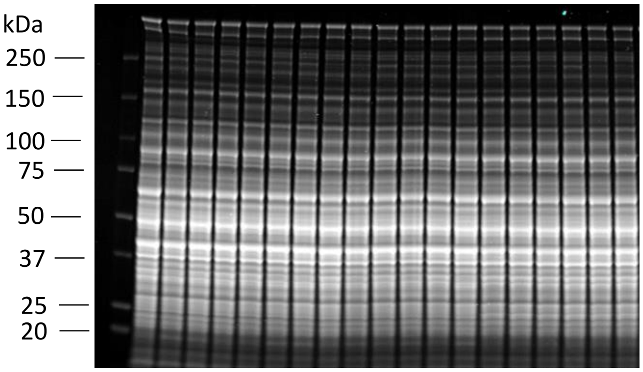

Samples are loaded in the same order as for western blotting.

Figure 3A

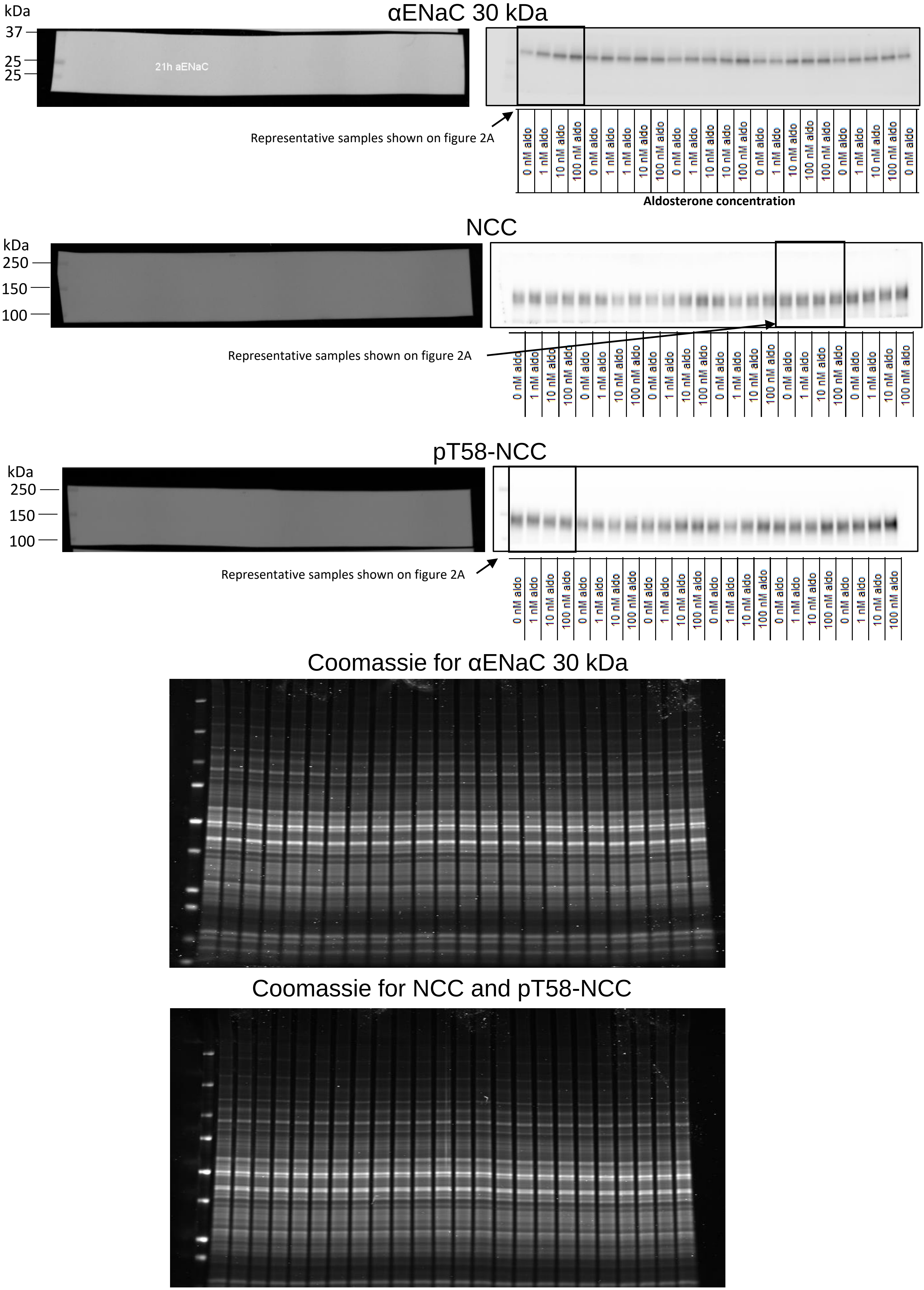
αENaC 30 kDa
kDa
37
25
25
21h aENaC
Representative samples shown on figure 2A
0 nM aldo 1 nM aldo 10 nM aldo 100 nM aldo 0 nM aldo 1 nM aldo 1 nM aldo 10 nM aldo 100 nM aldo 0 nM aldo 1 nM aldo 10 nM aldo 10 nM aldo 100 nM aldo 0 nM aldo 1 nM aldo 10 nM aldo 100 nM aldo 100 nM aldo 0 nM aldo 1 nM aldo 10 nM aldo 100 nM aldo 0 nM aldo
Aldosterone concentration
NCC
kDa
250
150
100
Representative samples shown on figure 2A
0 nM aldo 1 nM aldo 10 nM aldo 100 nM aldo 0 nM aldo 1 nM aldo 10 nM aldo 100 nM aldo 0 nM aldo 1 nM aldo 10 nM aldo 100 nM aldo 0 nM aldo 1 nM aldo 10 nM aldo 100 nM aldo 0 nM aldo 1 nM aldo 10 nM aldo 100 nM aldo 0 nM aldo 1 nM aldo 10 nM aldo 100 nM aldo
pT58-NCC
kDa
250
150
100
Representative samples shown on figure 2A
0 nM aldo 1 nM aldo 10 nM aldo 100 nM aldo 0 nM aldo 1 nM aldo 10 nM aldo 100 nM aldo 0 nM aldo 1 nM aldo 10 nM aldo 100 nM aldo 0 nM aldo 1 nM aldo 10 nM aldo 100 nM aldo 0 nM aldo 1 nM aldo 10 nM aldo 100 nM aldo 0 nM aldo 1 nM aldo 10 nM aldo 100 nM aldo
Coomassie for αENaC 30 kDa
Coomassie for NCC and pT58-NCC

Figure 3E

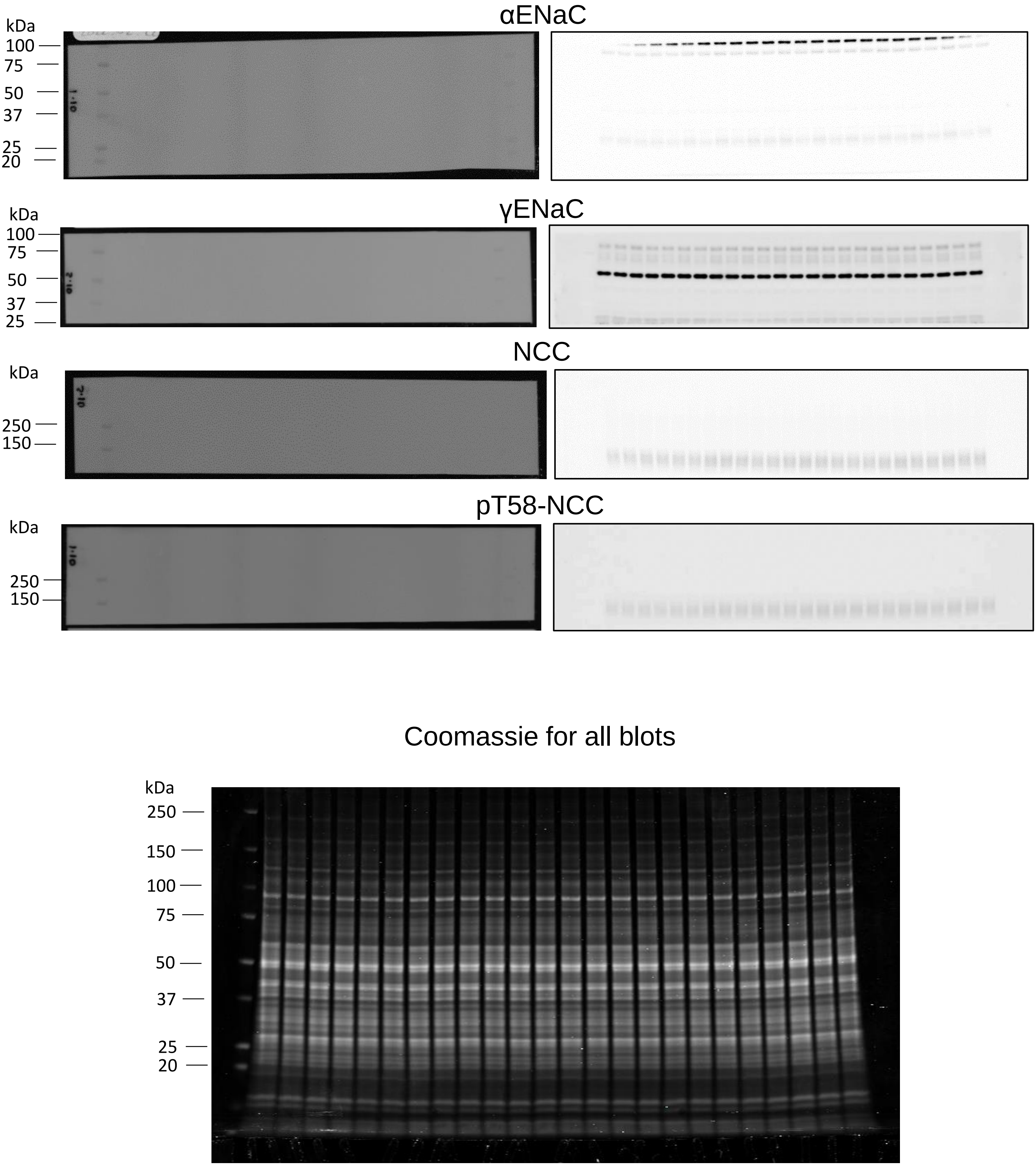
αENaC
kDa
100
75
50
37
25
20
γENaC
kDa
100
75
50
37
25
NCC
kDa
250
150
pT58-NCC
kDa
250
150
Coomassie for all blots
kDa
250
150
100
75
50
37
25
20

Supplemental Figure S5

αENaC 30 kDa

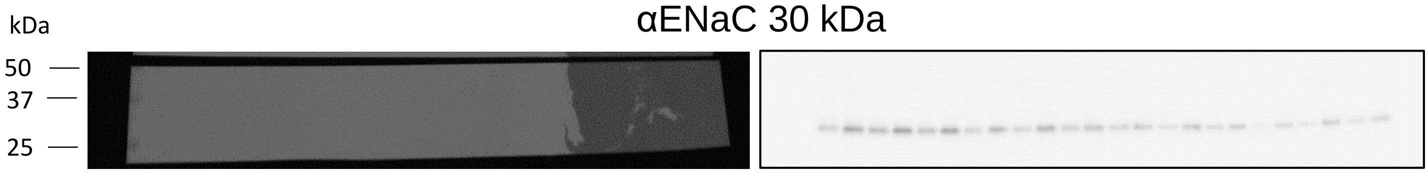

NCC

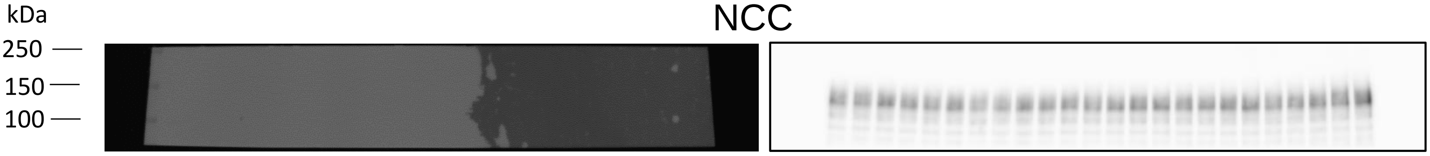

pT58-NCC

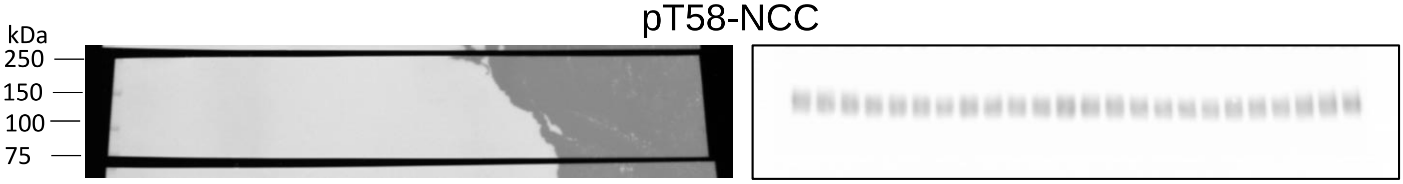

Coomassie for all blots

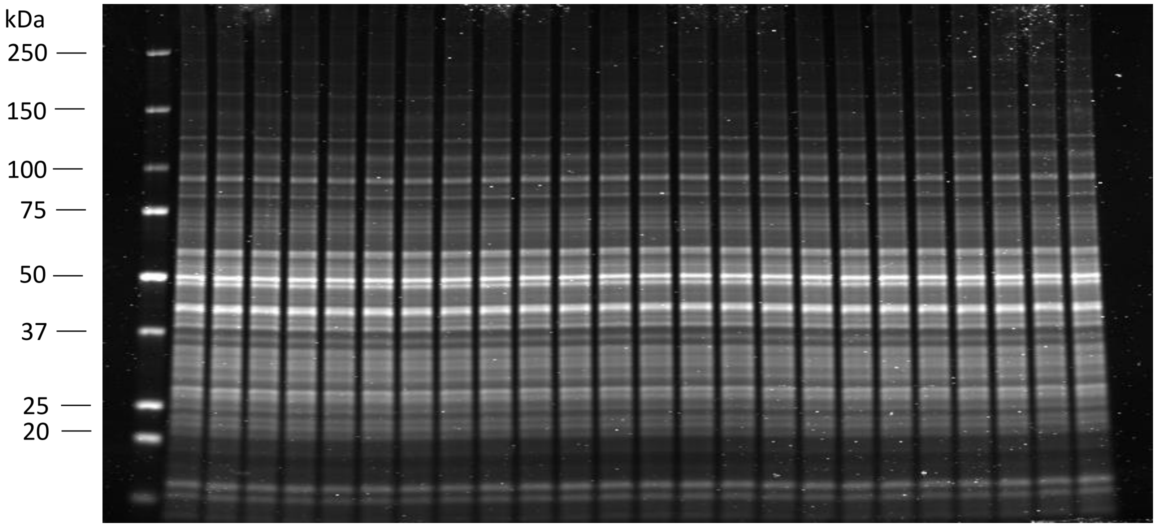

Figure 4A

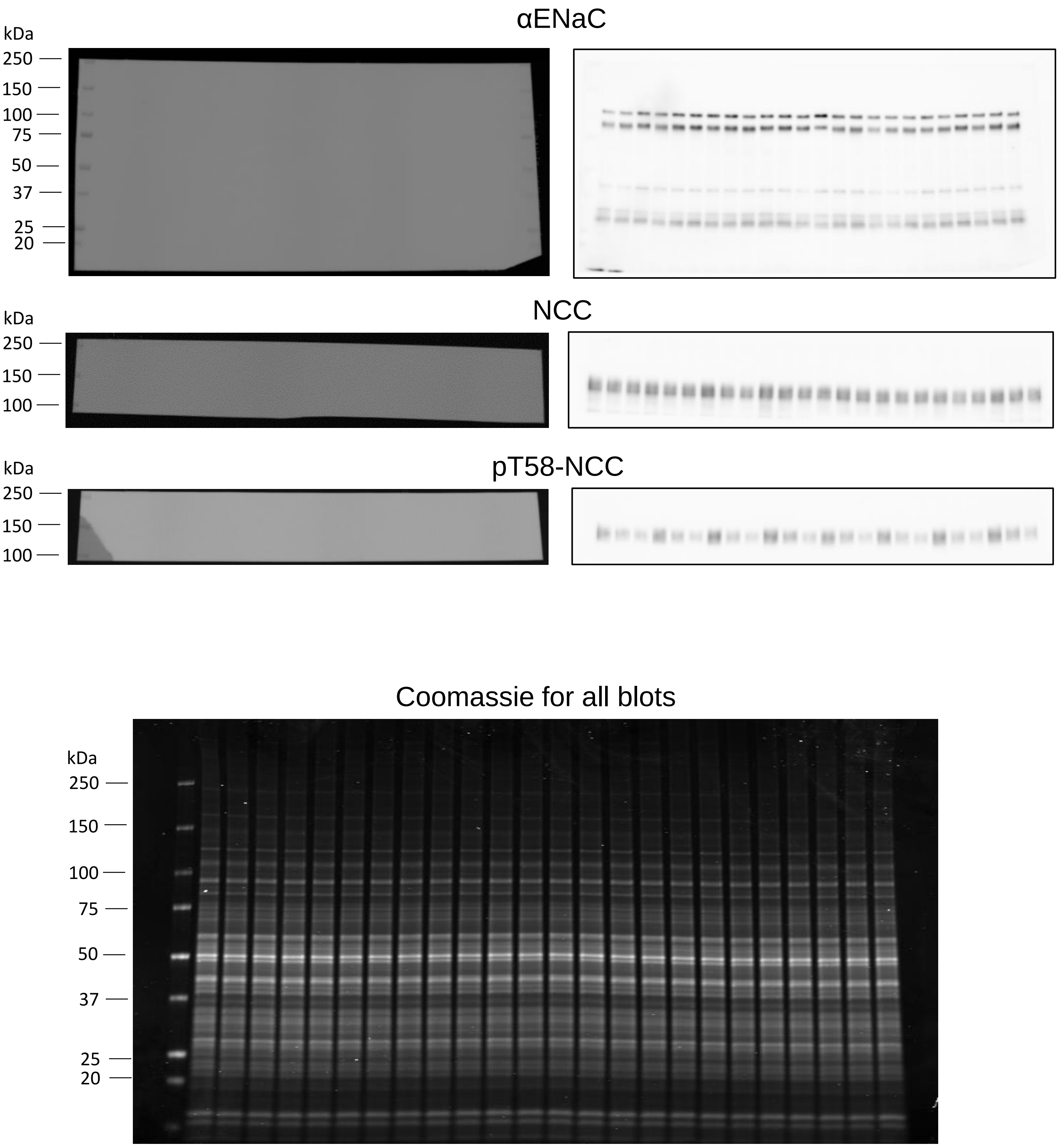
αENaC
kDa
250
150
100
75
50
37
25
20
NCC
kDa
250
150
100
pT58-NCC
kDa
250
150
100
Coomassie for all blots
kDa
250
150
100
75
50
37
25
20

Supplemental Figure S6

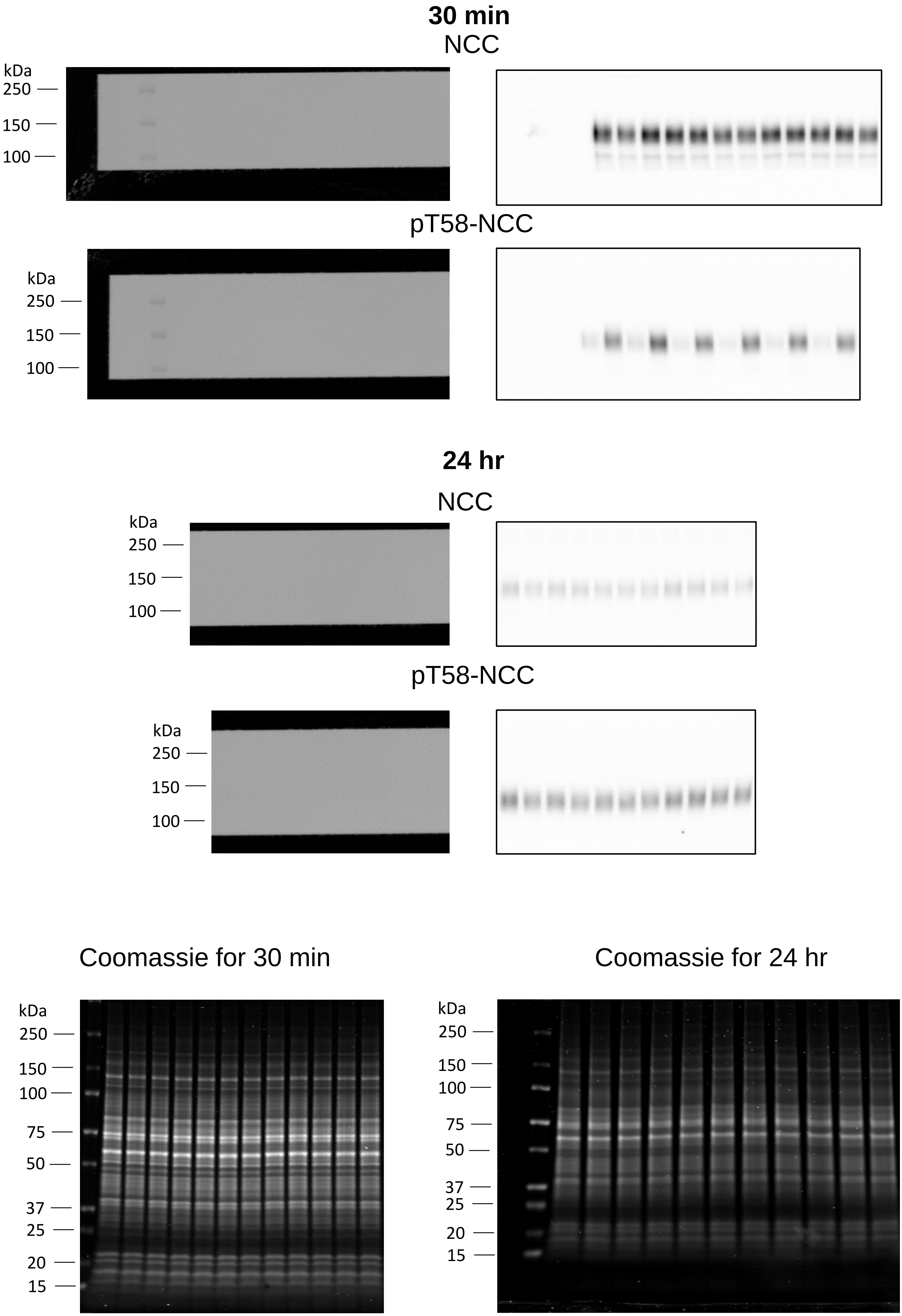

Supplement: Supplementary file 1 [file DataSheet1.PDF]
